# Interruption of aberrant chromatin looping is required for regenerating *RB1* function and suppressing tumorigenesis

**DOI:** 10.1038/s42003-022-04007-2

**Published:** 2022-09-29

**Authors:** Xuyang Wen, Tianyi Ding, Fang Li, Jiayan Fan, Xianqun Fan, Renbing Jia, He Zhang

**Affiliations:** 1 Institute for Regenerative Medicine, Shanghai East Hospital, Frontier Science Research Center for Stem Cells, School of Life Science and Technology, Tongji University, Shanghai, P. R. China; 2 Department of Ophthalmology, Shanghai Key Laboratory of Orbital Diseases and Ocular Oncology, Ninth People’s Hospital, Shanghai JiaoTong University School of Medicine, Shanghai, P. R. China

**Keywords:** Chromatin structure, Tumour-suppressor proteins

## Abstract

RB transcriptional corepressor 1 (*RB1*) is a critical regulatory gene in physiological and pathological processes. Genetic mutation is considered to be the main cause of *RB1* inactivation. However, accumulating evidence has shown that not all *RB1* dysfunction is triggered by gene mutations, and the additional mechanism underlying *RB1* dysfunction remains unclear. Here, we firstly reveal that a CCCTC binding factor (CTCF) mediated intrachromosomal looping served as a regulatory inducer to inactivate *RB1*. Once the core genomic fragment was deleted by Clustered Regularly Interspaced Short Palindromic Repeats/Cas9 (CRISPR/Cas9), this intrachromosomal looping was disrupted. After the open of chromatin, Enhancer of Zeste Homolog 2 (EZH2) was released and decreased the level of Tri-Methyl-Histone H3 Lys27 (H3K27me3) at the *RB1* promoter, which substantially restored the expression of RB protein (pRB) and inhibited tumorigenesis. In addition, targeted correction of abnormal *RB1* looping using the small-molecule compound GSK503 efficiently restored *RB1* transcription and suppressed tumorigenesis. Our study reveals an alternative transcriptional mechanism underlying *RB1* dysfunction independent of gene mutation, and advancing the discovery of potential therapeutic chemicals based on aberrant chromatin looping.

## Abstract (web summary)

A CTCF mediated intrachromosomal looping of *RB1* promoter and its downstream silencer served as a regulatory inducer to inactivate *RB1*, revealing a novel transcriptional mechanism underlying *RB1* dysfunction independent of gene mutation.

## Introduction

RB transcriptional corepressor 1 (*RB1*) was the first tumor suppressor gene to be found in 1971^1^. Its encoded protein has been proven to have a regulatory role in cell cycle transition, chromosome stability, and cellular metabolism^2^. Dysfunction of the *RB1* gene directly causes various pathological processes, including developmental deficit, immunological microenvironment disorder, and childhood cancer^3^. Genetic mutations were demonstrated to be the main reason for *RB1* inactivation^4^. According to the Retinoblastoma Gene Mutation Database (RBGMdb), a total of 3393 variants of *RB1* have been reported so far. However, with the increasing number of patient genomes being sequenced, ever-increasing evidence has shown that not all *RB1* dysfunction patients undergo *RB1* genetic mutation^5^. A report has revealed that many retinoblastoma patients do not have any *RB1* exon mutation, and from the point of view of the whole genome, the very few genetic mutations found may not be enough to induce tumorigenesis^6^. In addition, a family study of multiple myeloma did not detect any deletion of the *RB1* locus, which is frequently deleted in patients with multiple myeloma^7^. These studies raised the possibility that additional causes beyond traditional genetic mutations induce *RB1* dysfunction to mediate pathological processes.

Considering these matters as a whole, there is the fundamental fact that the human genome does not exist as a linear entity. The string of nucleotides is wrapped around histones and organized in three-dimensional (3D) space^8^. It is now clear that the process of gene transcriptional regulation is highly orchestrated by the 3D structure of chromosomes^9^. On the one hand, the formation of chromosomal looping between gene promoter and enhancer could activate gene transcription by recruiting the transcriptional activator^10^. On the other hand, chromosomal looping could also inhibit gene expression by recruiting transcriptional repressor^11^. Thus, the important function of chromosomal looping in regulating gene transcription was always illustrated in a cell-specific pattern^12–14^. We have highlighted the importance of the intrachromosomal loop as a critical epigenetic barrier in cell reprogramming and maintenance of genomic imprinting^15,16^. In addition, abnormal chromatin interactions have also been found to regulate the transcription of *PDGFRA, MYC,* and *FOXA1* genes in the tumorigenesis of glioma, acute myelogenous leukemia, and prostate cancer, respectively^17–19^. However, the role of 3D chromosome structure in *RB1* dysfunction remains an open question.

In this work, we hypothesized whether chromatin loops might have direct influence in *RB1* dysfunction and tumorigenesis. We revealed that the formation of a special chromosomal loop at the *RB1* locus was an alternative regulatory inducer of *RB1* dysfunction. In addition, we demonstrated that the small-molecule compound GSK503 efficiently restored pRB expression and suppressed tumorigenesis via targeted correction of abnormal *RB1* chromatin looping. These results suggested an alternative transcriptional mechanism underlying *RB1* dysfunction independent of gene mutation, and discovered efficient chemical compounds by targeting aberrant chromatin looping, thereby providing an alternative avenue for exploring gene dysfunction without mutation, and advancing the discovery of therapeutic chemicals based on chromatin looping.

## Results

### A functional intrachromosomal looping at *RB1* locus

**Fig. 1:**
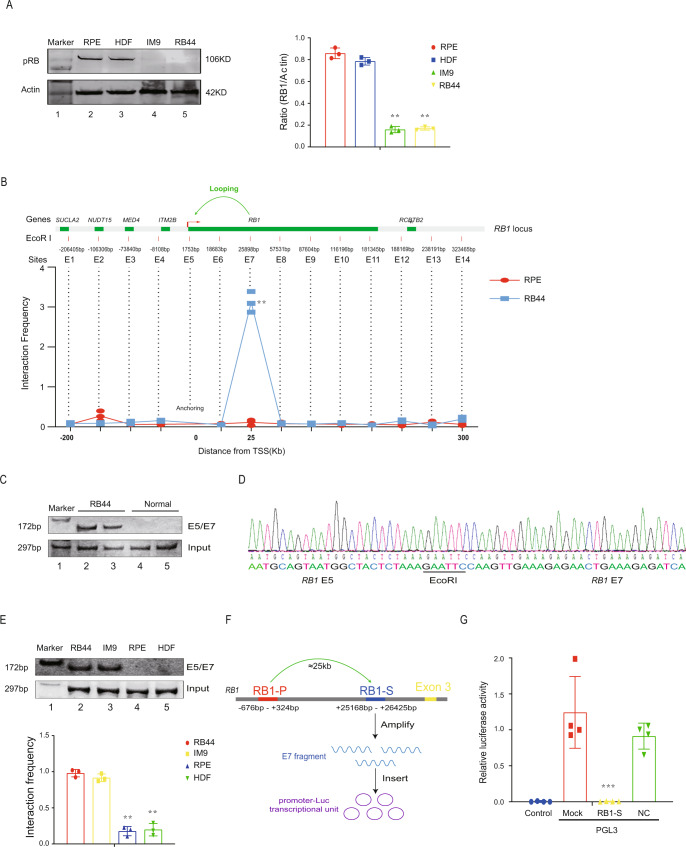
A functional chromosomal looping at *RB1* locus. **A** Western blot showed abundance of pRB at the protein level in RB44, IM9, and the control (RPE and HDF) cell lines. **P < 0.01. **B** A chromosomal conformation capture (3C) assay was performed to detect intrachromosomal interactions between the *RB1* promoter and regions in the *RB1* locus. Top: Schematic diagram of variant primer sets in 3C assay. Using *Eco*RI as the enzyme cutting site, 14 sites were selected around the 13q14 locus. E5 was set as 3C bait. Bottom: the intrachromosomal interaction frequency between the E5 and E7 regions was determined by normalizing the 3C PCR signal to that of the positive control (input DNA). **P < 0.01 compared to negative control ARPE19 cells. **C** A 3C assay was performed to detect the chromosomal looping between E5 and E7 regions in RB44 and the control (RPE) cells by PCR. **D** The 3C products were confirmed by DNA sequencing. The 3C products derived from the *RB1* promoter E5-E7 interaction were cloned and sequenced. The 3C products contained the *Eco*RI site that was flanked on both sides near the TSS and 25 kb downstream. We set the TSS of *RB1* gene as Zero Point, the distance between the TSS of *RB1* and each *Eco*RI cutting site was shown in (**B**). E5 1753 bp: the distance between the TSS of *RB1* and E5 *Eco*RI cutting site. E7 25898 bp: the distance between the TSS of *RB1* and E7 *Eco*RI cutting site. **E** A 3C assay was performed to detect the existence of E5-E7 chromosomal looping in IM9 and the control (HDF) cells by PCR. **P < 0.01. **F** Schematic of pGL3-promoter-RB1-S (pGL3-*RB1-S*) construction. The E7 fragment was amplified and inserted into pGL3-promoter-Luc. RB1-P, promoter of RB1; RB1-S, suppressor of RB1. **G** The promoter activity detected in dual luciferase reporter system. The 1.2 kb E7 fragment was amplified and inserted into pGL3-promoter-Luc with firefly luciferase reporter (pGL3-RB1-S). The similar random fragment was amplified and inserted into pGL3-promoter-Luc for negative control (pGL3-NC). Empty pGL3-promoter-Luc vector was used as mock. All the above three groups were transfected into pRL-TK vector with renilla luciferase, which was used as internal control to detect the transfection efficiency. Control: cells were transfected with pRL-TK vector only. All data were calculated as the ratio of firefly to renilla luciferase activity (Fluc/Rluc) in dual luciferase reporter system. For comparison, the ratio of Fluc/Rluc of the mock was arbitrarily set as 1 in the calculation. ***P < 0.001 compared to mock luciferase expression.

We chose two cell lines, including the retinoblastoma cell line RB44 (Supplementary Data 1) and the human multiple myeloma cell line IM9^20^ (Cancer Cell Line Encyclopedia, CCLE, https://sites.broadinstitute.org/ccle, COSMIC ID: 753563), as the non-mutation model of *RB1* dysfunction. As expected, RB44 and IM9 cells showed minimal expression of pRB, and sequencing of all *RB1* exons did not detect any mutations (Fig. 1A, Supplementary Table 1), which allowed us to explore the cause of *RB1* inactivation without interference from genetic mutations. According to the peaks of protein binding (CTCF, H3K27me3, and H3K4me3) in UCSC genome browser (Supplementary Fig. 1), we designed 3C assay to examine chromatin interaction across the entire *RB1* locus in 13q14. Interestingly, the quantitative results showed that the *RB1* promoter region (site E5) interacted frequently with a chromatin region of the *RB1* intron (site E7) in RB44 cells (Fig. 1B; Fig. 1C, lanes 2–3), while this chromatin loop was rarely detected in normal RPE cells (Fig. 1B; Fig. 1C, lanes 4–5). Although we also found additional chromatin loop in HDF (human fibroblasts) control cells in intron 17 adjacent to exon 18 of the *RB1* gene, but not in RB44 cells and retinoblastoma-specific RPE control cells (Supplementary Fig. 2), we thus focused on the E5-E7 looping. DNA sequencing verified the existence of this E5-E7 loop between the *RB1* promoter (*RB1-P*, site E5) and site E7 (~25.8 kb downstream of the *RB1* transcriptional start site (TSS) and ~24 kb downstream of E5 site) (Fig. 1D). Similar to RB44, this chromatin loop was also found in IM9 cells (Fig. 1E, lane 3). To address the transcriptional activity of the E7 DNA fragment, we cloned a 1.2-kb fragment encompassing the E7 site into a dual luciferase reporter gene system (Fig. 1F). As shown in Fig. 1G, the 1.2-kb *RB1-S* DNA fragment significantly reduced luciferase activity, while negative control with random fragment and mock control with empty vector did not change the luciferase activity. These results showed that E7 region might function as a potential gene suppressor and the *RB1-P-S* chromatin loop could be a regulatory factor that induces *RB1* dysfunction in tumors.

### Interruption of chromosomal looping suppresses tumorigenesis

**Fig. 2:**
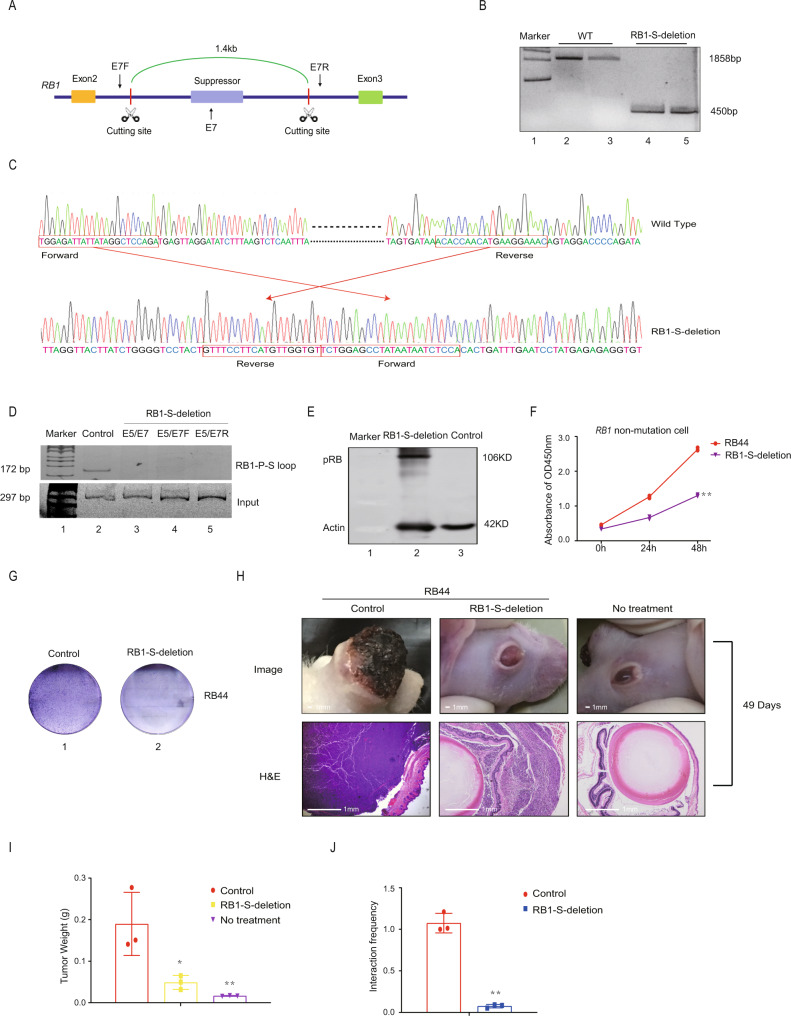
Interruption of the chromosomal looping suppresses tumorigenesis. **A** Schematic of the *RB1* suppressor fragment deletion by CRISPR-Cas9. **B**, **C** PCR and sequencing of the deletion of the 1.4-kb suppressor region from the genome by CRISPR-Cas9. WT, wild-type RB44 cells; *RB1-S*-deletion, 1.4-kb suppressor region deleted RB44 cells. E7F and E7R primers were used. **D** PCR was performed to test the *RB1-P-S* loop after *RB1-S* deletion. Primer pairs E5/E7, E5/E7F, and E5/E7R were used, respectively. **E** The Western blot analysis showed the expression of *RB1* at protein level after *RB1-S* deletion. **F** An in vitro cell proliferation assay carried out by a cell counting kit showed the proliferation ability in *RB1-S* deletion RB44 cells and wild-type RB44 cells. **P < 0.01. **G** A soft agar tumor formation assay was performed to determine the colony formation ability of *RB1-S*-deleted RB44 cells and wild-type RB44 cells. **H** Top: General photograph of orthotopic xenograft at 49 days after implantation by the injection of RB44 cells into the vitreous with or without *RB1-S* deletion; Bottom: representative images of H&E staining for the evaluation of tumor formation. *n* = 5. Scale bars: 1 mm. **I** The suppressive effects of *RB1-S* deletion on tumor weight in a subcutaneous xenograft model. *P < 0.05, **P < 0.01. **J** PCR was performed to determine the *RB1-P-S* loop in the *RB1-S* deleted xenograft tissues. **P < 0.01.

To investigate the role of the *RB1-P-S* loop in *RB1* dysfunction and tumorigenesis, we then deleted the *RB1-S* region using the CRISPR/Cas9 system (Fig. 2A). As shown in Fig. 2B, *RB1-S* was completely deleted from the genome, and we further verified this deletion by sequencing (Fig. 2C, Supplementary Table 4). Next, we found that the *RB1-P-S* loop was not detected with primers inside (E7) or outside (E7F and E7R) the deleted region in the *RB1-S*-deleted cells (Fig. 2D, lanes 3–5), whereas it was detected in the non-*RB1-S*-deleted cells (Fig. 2D, lane 2; Supplementary Fig. 2). More importantly, we observed that pRB expression at the protein level was restored in the *RB1-S*-deleted cells (Fig. 2E). These data showed that a lack of the *RB1-P-S* loop was required for *RB1* activation. We then asked whether the abolishment of the *RB1-P-S* loop was beneficial to tumor suppression. We carried out in vitro cell proliferation assays and in vivo orthotropic xenograft experiments. As expected, cell proliferation was significantly reduced after the disruption of the *RB1-P-S* loop (Fig. 2F, G). In the orthotopic xenograft model of nude mice, we noticed that the lack of the *RB1-P-S* loop resulted in a significant reduction in tumor volume compared to that of the wild-type mice (Fig. 2H). By weighing the eyeball tissue, we then found that the *RB1-S*-deleted group showed an ~50% decrease in tumor weight compared with the wild-type group (Fig. 2I). In xenograft tissues, we confirmed that the *RB1-P-S* loop was not detected in the *RB1-S*-deleted groups (Fig. 2J). Taken together, these results demonstrated that the *RB1-P-S* loop was a potential regulatory target that induced *RB1* dysfunction and triggered tumorigenesis.

### CTCF-PRC2 complex is involved in the chromosomal looping at *RB1* locus

**Fig. 3:**
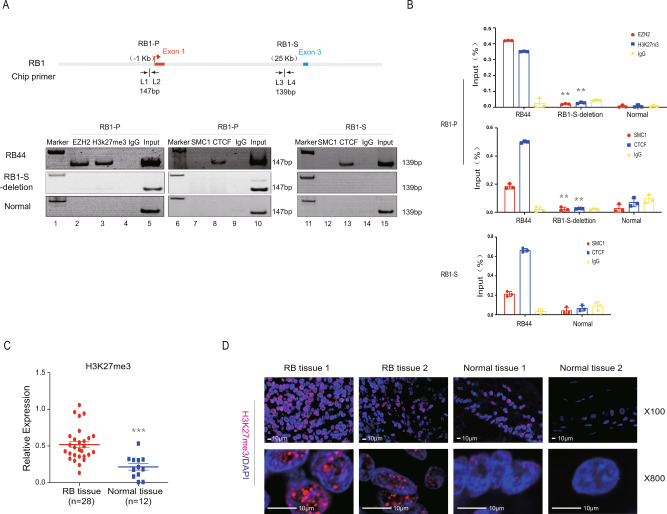
CTCF-PRC2 complex is involved in the chromosomal looping at *RB1* locus. **A** Top: Schematic of the ChIP site. L1-L4: primers in *RB1* promoter region and *RB1* suppressor region. Bottom: ChIP assay of the EZH2, H3K27me3, CTCF, and SMC1 status in the *RB1* promoter region and the *RB1* suppressor region. Normal: RPE cells. **B** The quantified ChIP was further confirmed by the above results. **P < 0.01. **C** H3K27me3 expression in retinoblastoma tissues (*n* = 28) compared with normal eye tissues (*n* = 12). ***P < 0.001. **D** Immunofluorescence assays were performed with H3K27me3 probes in retinoblastoma tissues and normal tissues. Scale bars: 10 µm.

Next, we tried to identify a suitable molecular compound for targeted disruption of the *RB1-P-S* loop and reactivation of the *RB1* gene. One possible strategy was to unlock the *RB1-P-S* loop by determining the core modified factors. We then examined the core factors for organizing the *RB1-P-S* chromatin loop. CTCF participates in the formation of chromatin loop by directional binding to CTCF binding sites (CBSs) by its 11 zinc fingers^21–23^. We firstly analyzed the location and orientation of CBSs in the E5 promoter region and E7 silencer region, respectively. With bioinformatic program^21^, we found that six forward CBSs were located in the E5 region and other five reverse CBSs located in the E7 region (Supplementary Fig. 3). Next, we found that CTCF could directly bind to the promoter and suppressor of *RB1* (Fig. 3A, first panel, lanes 8 and 13). Since CTCF could contribute to the formation of chromosomal looping by interacting with Cohesin^14,24^, we then focused on the SMC1, a core protein of Cohesin complex. To further determine whether SMC1 was involved in the formation of *RB1-P-S* looping, we examined the formation of *RB1-P-S* looping by silencing of SMC1 (Supplementary Table 4). After SMC1 knockdown (Supplementary Fig. 4a, b), the formation of *RB1-P-S* looping was not changed (Supplementary Fig. 4c, lanes 3–4). These data suggested that SMC1 was not involved in the formation of *RB1-P-S* loop.

Given the fact that CTCF has been found to interact with PRC2 complex, which could regulate trimethylation level of H3K27 (H3K27me3). We firstly focused on the level of H3K27me3 at the *RB1* promoter. We found a stronger signal of the level of H3K27me3 at the *RB1* promoter in tumor cells (Fig. 3A, first panel, lane 3). After *RB1-S* deletion and *RB1-P-S* loop disruption, chromatin immunoprecipitation (ChIP) data showed that the H3K27me3 level of the *RB1* promoter was markedly decreased (Fig. 3A, second panel, lane 3). We further showed the similar results by using ChIP with quantitative real-time PCR (ChIP-qPCR) (Fig. 3B). To verify these results, we used a tissue chip containing 28 retinoblastoma tissues and 12 normal eye controls, and we found a prominent increase of H3K27me3 level in retinoblastoma tissues compared with normal eye tissues (Fig. 3C). Immunofluorescence assays confirmed the strong staining of H3K27me3 in retinoblastoma tissues (Fig. 3D). These data showed that the decrease of H3K27me3 modification at *RB1* promoter was caused by interruption of *RB1-P-S* chromatin loop. Since the EZH2 protein, a core component of the PRC2 complex, has been shown to control the level of H3K27me3^25^, we next focused on EZH2 binding at the *RB1* promoter. Similarly, we found that deletion of the *RB1-S* region abolished EZH2 binding at the *RB1* promoter in *RB1-S*-deleted cells compared to wild-type tumor cells (Fig. 3A, second panel, lane 2). Given the above results, it raised a possibility that inhibition of EZH2 might reactivate the pRB expression by the decrease of H3K27me3 level at *RB1* promoter through using EZH2 inhibitor.

### Silencing of CTCF abolishes the chromosomal looping and reactivates pRB expression

**Fig. 4:**
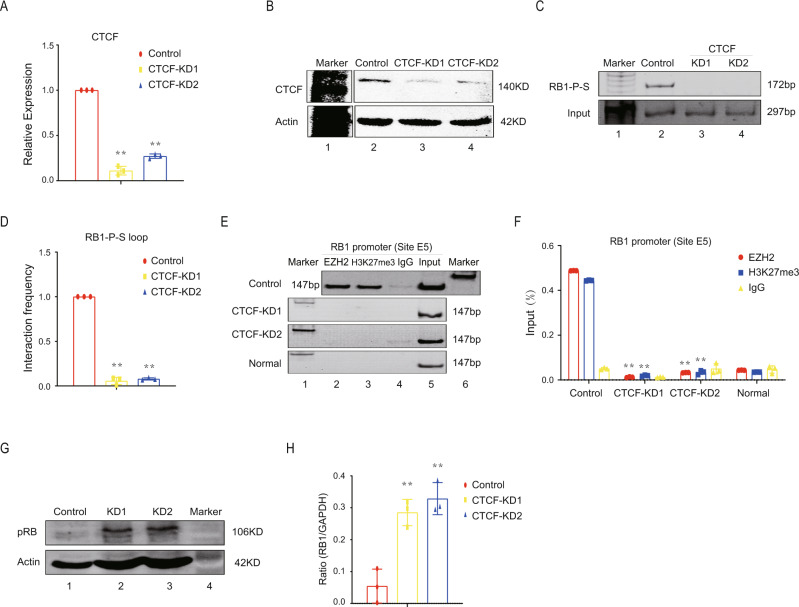
Silencing of CTCF abolishes the chromosomal looping and reactivates pRB expression. **A** RT-qPCR of CTCF expression at RNA level after CTCF knockdown with shRNAs transfection. **P < 0.01. **B** Western blot was performed to detect CTCF protein expression after shRNA transfection. **C**, **D** 3C assay of the existence of chromosomal looping after CTCF knockdown by PCR and qPCR, respectively. **P < 0.01. **E**, **F** ChIP assay of the binding of EZH2 to the *RB1* promoter (site E5) and the level of H3K27me3 after CTCF knockdown. **P < 0.01. **G**, **H** Western blot of the expression level of pRB after CTCF knockdown. **P < 0.01.

To determine the critical role of CTCF in orchestrating the intrachromosomal looping at *RB1* locus, we knocked down CTCF in *RB1* non-mutation cells by using conventional shRNA method. As expected, we found that the *CTCF* expression was significantly reduced in real-time PCR assay (Fig. 4A). Moreover, western-blot assay further confirmed that the protein level of CTCF was remarkably decreased as compared to control (Fig. 4B, lanes 2–3). Next, 3 C assay showed that silencing of CTCF abolished the *RB1-P-S* intrachromosomal looping between *RB1* promoter and suppressor (Fig. 4C, lanes 3–4; Fig. 4D). After CTCF knockdown, the ChIP assay also showed that the binding of EZH2 to the *RB1* promoter was decreased (Fig. 4E, lane 1), which led to a decrease in H3K27me3 in the *RB1* promoter (Fig. 4F). To further determine whether this intrachromosomal looping was responsible for *RB1* silencing, we detected the expression level of *RB1* in CTCF silencing group. As expected, the expression of pRB was significantly reactivated once CTCF was silenced (Fig. 4G, H). These data showed that the abolishment of the abnormal intrachromosomal loop was critical cause for reactivating of pRB expression.

### GSK503 interrupts the chromosomal looping and restores pRB expression in *RB1* non-mutated tumor cells

**Fig. 5:**
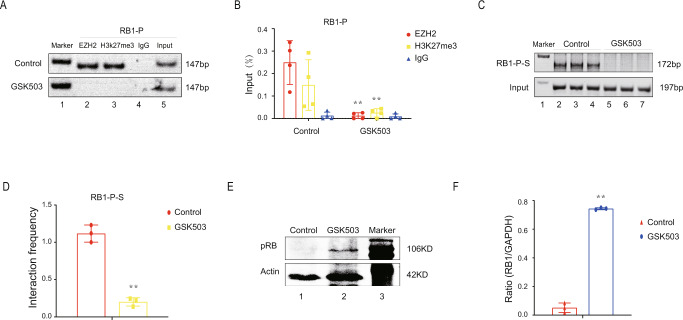
GSK503 interrupts the chromosomal looping and restores pRB expression. **A**, **B** ChIP assay of the binding of EZH2 to the *RB1* promoter (site E5) and the level of H3K27me3 after GSK503 treatment. **P < 0.01. **C**, **D** 3C assay of the *RB1-P-S* chromosomal loop after GSK503 treatment. **P < 0.01. **E**, **F** Western blot of pRB expression after GSK503 treatment. **P < 0.01.

To test this hypothesis, we carried out a preclinical study using an EZH2 inhibitor, the leading molecular compound GSK503, which is a small chemically optimized compound that efficiently targets the SET domain of EZH2 and exhibits favorable pharmacokinetics in mice. EZH2 inhibitor has been reported to be efficient efficacy in *RB1*-mutational Y79 and Weri retinoblastoma cells^26^. We next treated *RB1* non-mutated tumor cells with 10 µM GSK503 for 72 h and found that the binding of EZH2 to the *RB1* promoter was abolished, which led to a decrease in H3K27me3 in the *RB1* promoter (Fig. 5A, B). As expected, GSK503 caused the targeted disruption of the *RB1-P-S* loop between the *RB1* promoter and suppressor (Fig. 5C), and none of the treatment groups retained the *RB1-P-S* loop (Fig. 5D). Most importantly, after GSK503 treatment, the protein expression of *RB1* was significantly restored (Fig. 5E, F). These results suggested that GSK503 was an efficient chemical compound to realize the targeted disruption of the CTCF-EZH2 mediating chromatin loop by inhibiting EZH2 activity and thereby to reactivate the *RB1* gene in tumor cells without *RB1*-mutations.

### GSK503 obtains efficient therapeutical effect in *RB1* non-mutated tumors

**Fig. 6:**
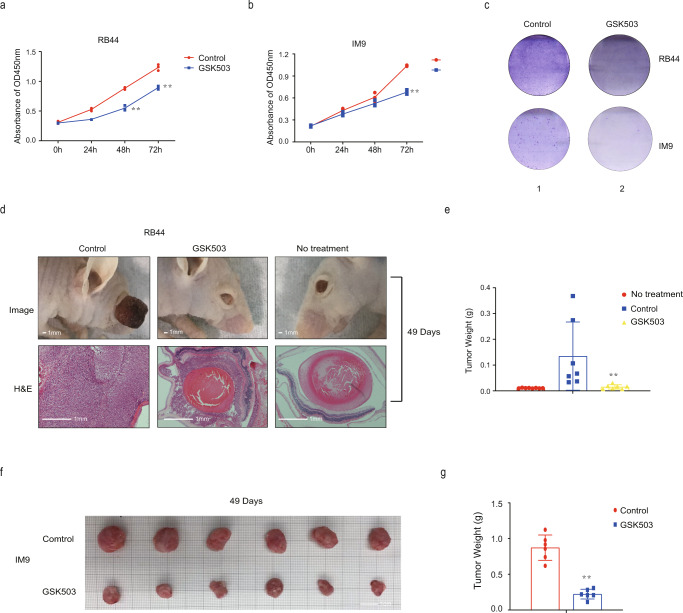
GSK503 obtains efficient therapeutical effect in *RB1* non-mutated tumors. **a**, **b** The proliferation ability of RB44 and IM9 cells after GSK503 treatment. **P < 0.01. **c** A soft agar colony formation assay was performed to determine the tumor formation ability of RB44 and IM9 cells after GSK503 treatment. **d** Suppressive effects on tumor volume in the GSK503-treated group in the orthotopic xenograft model. Scale bars: 1 mm. **e** Suppressive effects on tumor weight in the GSK503-injected group in the orthotopic xenograft model. **P < 0.01. **f** Suppressive effects on tumor volume in the GSK503-injected group in the subcutaneous xenograft model. **g** Suppressive effects on tumor weight in the GSK503-injected group in the subcutaneous xenograft model. **P < 0.01.

Next, we examined the role of GSK503 in *RB1* non-mutated tumor cells. As expected, the cell proliferation assay showed that GSK503 significantly reduced tumor proliferation and colony formation in both RB44 and IM9 tumor cells in vitro (Fig. 6a–c). Next, we tried to verify this outcome in vivo. An orthotopic xenograft model of retinoblastoma cells was used. After the tumors formed one week after orthotopic transplantation, a daily subconjunctival dose of 3 µl of 10 µM GSK503 was administered for 7 days. The mice treated with GSK503 exhibited a significant reduction in tumor size. H&E staining also showed that the intraocular structure in the non-GSK503-treated group was destroyed, while tumor proliferation in the GSK503-treated group was suppressed and the intraocular structure was retained (Fig. 6d). We also noticed that GSK503 significantly reduced the tumor weight (Fig. 6e). A heterotopic transplantation model was used for IM9 cells. Subcutaneous injection of 3 µl of 10 µM GSK503 was carried out for 7 days once the tumor formed, and the GSK503-treated group showed significant tumor suppression (Fig. 6f). By weighing the tumor tissues, we observed that GSK503 significantly reduced the tumor weight (Fig. 6g). Together, these results reveal that GSK503 has efficient therapeutic efficacy in restored pRB expression and suppressed tumorigenesis.

## Discussion

**Fig. 7:**
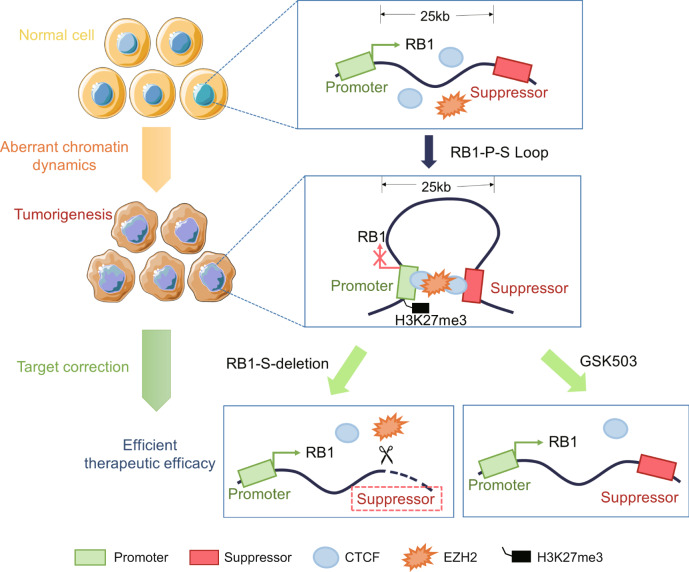
Schematic diagram of the research model. When the CTCF-mediated chromosomal loop between the *RB1* promoter and its 25 kb downstream suppressor was formed, EZH2 was then recruited and increased the level of H3K27me3 at the *RB1* promoter, thereby inhibiting pRB expression and leading to tumorigenesis. The targeted correction of abnormal chromosomal interactions inhibited tumorigenesis by suppressor deletion or GSK503 inhibition. Every element of this image is new-created by the authors.

As a major factor influencing gene dysfunction, genetic mutation is generally considered to be the trigger of gene dysfunction^27^. However, with the development of high-throughput sequencing technology, increasing evidence has shown that genetic mutations are not the only cause of gene dysfunction^6^. The occurrence of acute senescence is mediated by non-mutated *HMGA2* dysfunction^28^. *BRCA1* is an important tumor suppressor, and 35.3% of *BRCA1* dysfunction in ovarian cancer was found to be unrelated to germline or somatic mutations^29^. Dysfunction of *TP53* without genetic mutations in tumorigenesis, cell cycle arrest, apoptosis, and senescence has also been reported^30^. Gene mutation was found to be the major cause of biallelic inactivation of *RB1*^31^; however, the additional mechanism underlying *RB1* dysfunction remains unknown^32^. In this study, to the best of our knowledge, we demonstrate for the first time that the formation of a special intrachromosomal loop at the *RB1* locus is an alternative cause of *RB1* dysfunction. When the *RB1-P-S* chromosomal loop between the *RB1* promoter and suppressor was established, EZH2 was then recruited and increased the level of H3K27me3 at the *RB1* promoter, thereby inhibiting pRB expression and leading to tumorigenesis (Fig. 7).

Since *RB1* dysfunction without genetic mutation often induces the occurrence of disease by regulating various pathological processes^33^, our finding of the *RB1* chromosomal loop ushers in an interesting era of exploring the mechanisms of *RB1* dysfunction in these additional pathological processes. Most importantly, the dysfunction of many important physiological and pathological genes, including *PP2A* and *FOXG1*, is not always triggered by genetic mutations^34,35^. This study is likely to open a avenue for discovering the mechanisms underlying gene dysfunction without genetic mutations. It should be noted that a retroposon involved in the imprinting of the *RB1* gene in close proximity to our deleted region^36^. It raise an interesting possibility that the retroposon transcription may involve in *RB1* gene activation by reducing compaction of the nucleosomes at *RB1* promoter. Thus, it would be of great interest to explore this alternative mechanism of *RB1* activation.

It should be emphasized that this an alternative epigenetic model of pRB restoration is shown in non-*RB1*-mutational tumor cells in our study. However, it is unclear whether a similar epigenetic model is occurred in *RB1*-mutational tumor cells, and it would be of great interest to further explore the epigenetic possibility of *RB1* activation in *RB1*-mutational tumor cells. An alternative explanation of pRB epigenetic restoration in non-*RB1*-mutational tumor cells is that the restoration of pRB expression is likely to require two-layer gene regulation, including firstly correct chromosomal looping for epigenetically *RB1* activation and secondly correct *RB1* exon sequences for genetically accurate pRB translation. Therefore, the abolishment of aberrant chromosomal looping was enough to make sure the restoration of pRB expression, because correct sequences of *RB1* exons in non-*RB1*-mutational tumor cells would not result in inaccurate pRB translation. Moreover, in terms of epigenetics of *RB1* regulation in tumors, CTCF firstly orchestrates a promoter-enhancer chromosomal looping and then recruits EZH2 to change H3K27me3 modification, which subsequently co-establish the regulatory pathway and suppress the pRB expression. However, either the interruption of RB1-P-S looping or the abolishment of EZH2 by GSK503 will destroy the epigenetic pathway of pRB suppression and reactivate pRB expression. Further studies should focus on the identification of unknown factors involved in this epigenetic regulatory process of pRB expression.

As a key part of epigenetic studies, abnormal chromosomal conformation has been proven to play an important role in several physiological and pathological processes^37^. Our previous study highlighted the importance of the intrachromosomal loop as a critical epigenetic barrier in cell reprogramming^16^. The formation of an abnormal chromatin loop activated the oncogene *PDGFRA* and triggered tumorigenesis^17^. Effective targeted correction of these abnormal chromatin loops is an open question and a research hotspot. Most current studies focus on deleting abnormal genomic fragments or ectopically expressing foreign factors^38^; however, these classic experimental technologies in the laboratory have obvious limitations for further industrial application. Finding suitable leading small-molecule compounds for chromatin correction is an efficient and promising way to overcome these barriers^6^. This is, to our knowledge, the first study showed that the small-molecule compound GSK503 efficiently restored pRB expression and suppressed tumorigenesis in non-*RB1*-mutational tumor cells. Although targeted correction of abnormal *RB1* chromatin looping is one of the causes in the GSK503 mediating tumorigenesis suppression, we cannot eliminate other genetic or epigenetic causes. Thus, it would be of great interest to focus on the identification of other affects to better understand the role of EZH2 inhibitor in suppressing tumorigenesis. Most importantly, many physiological and pathological genes are regulated by alteration of chromosomal conformation. This finding will advance the discovery of useful chemical compounds by directing focus to the targeted correction of aberrant chromatin architecture, thereby accelerating the potential industrial and clinical prospects.

## Methods

### Cell culture

The *RB1* non-mutated human retinoblastoma cell line RB44 and human multiple myeloma cell line IM9 were cultured in RPMI-1640 (Gibco, USA) supplemented with 10% certified heat-inactivated fetal bovine serum (FBS; Gibco, USA), penicillin (100 U/ml), and streptomycin (100 μg/ml) at 37 °C in a humidified 5% CO_2_ atmosphere. The human normal cell lines HDF and RPE were cultured in DMEM (Gibco, USA) supplemented with 10% FBS (Gibco, USA), penicillin (100 U/ml), and streptomycin (100 μg/ml) at 37 °C in a humidified 5% CO_2_ atmosphere. IM9 and RPE cell lines were purchased from ATCC, RB44 and HDF were primary culture cells in our lab.

### RNA isolation and cDNA synthesis

Total RNA was extracted from cultured cells using TRIzol reagent (Sigma-Aldrich). The purified RNA was quantified using a Nanodrop 2000 UV-Vis Spectrophotometer (Thermo Scientific). The same amounts of total RNA were reverse transcribed into cDNA using the PrimeScript RT reagent Kit (TaKaRa Biotechnology).

### qRT-PCR

We validated the expression level of *RB1* by quantitative real-time RT-PCR (qRT-PCR). Expression levels were validated by real-time PCR using an ABI Prism 7500 (Applied Biosystems) using Power SYBR Green PCR Master Mix (Applied Biosystems) with the following protocol: an initial 10 min incubation at 95 °C followed by 40 cycles of 95 °C for 15 s and 60 °C for 30 s. All of the genes were normalized to the control gene *glyceraldehyde phosphate dehydrogenase (GAPDH)*.

### Chromosome conformation capture (3C)

1.0 × 10^7^ cells were cross-linked with 2% formaldehyde and quenched with 0.125 M glycine. The cells were lysed with cell lysis buffer (10 mM Tris [pH 8.0], 10 mM NaCl, 0.2% NP-40, and protease inhibitors), and the nuclei were collected. The nuclei were resuspended in 1× restriction enzyme buffer in the presence of 0.3% sodium dodecyl sulfate (SDS) and incubated at 37 °C for 1 h. Triton X-100 was then added to a final concentration of 1.8% to sequester the SDS. An aliquot of nuclei (2 × 10^6^) was digested with 800 U of the restriction enzyme *Eco*RI at 37 °C overnight. Then, 1.6% SDS was added, and the mixture was incubated at 65 °C for 20 min to stop the reaction. Chromatin DNA was diluted with T4 ligation buffer, and 2 μg DNA was ligated with 4000 U of T4 DNA ligase (Takara, Japan) at 16 °C for 4 h (final DNA concentration, 2.5 μg/ml). After treatment with 10 mg/ml proteinase K at 65 °C overnight to reverse the cross-links and with 0.4 μg/ml RNase A for 30 min at 37 °C, DNA was extracted with phenol-chloroform, ethanol precipitated, and used for PCR amplification of the ligated DNA products. We used nested PCR to achieve a final product of 100–350 bp in length for further analysis. The products of first PCR reaction were diluted to 1/100 to be the template of the second PCR reaction. First and second PCR cycles: an initial 2 min incubation at 95 °C followed by 30 cycles of 95 °C for 15 s, 65 °C for 30 s, and 72 °C for 30 s. The detailed PCR primers were shown in Supplementary Tables 2, 3.

### Luciferase assay

Site B4 and site B8 were amplified from genomic DNA using primers incorporating restriction enzyme sites (*Hin*dIII–*Xho*I) and then cloned into the *Hin*dIII–*Xho*I sites upstream of the promoter-*Luc* transcriptional unit. Luciferase assays were performed in 24-well white plates using the Luciferase Assay System (Promega, USA) according to the manufacturer’s protocol.

### ChIP

The ChIP assay was performed as previously described. The cells were fixed with 1% formaldehyde and centrifuged, and the pellets were resuspended in ChIP lysis buffer (50 mM Tris-HCl [pH 8.0]), incubated for 10 min on ice, and then sonicated (10 s on, 15 s off, output 30%, 4 min). The supernatant was collected into a new tube, and 5 mg of antibody (CTCF 1:200, EZH2 1:200, and H3K27 1:200 [Cell Signaling Technology], IgG 1:200 [Abcam]) was added. The mixture was then incubated overnight at 4 °C, and 60 ml of Pure Proteome Protein A and Protein G Magnetic Beads (Millipore) was used to pull down the DNA-protein-antibody complexes at 4 °C for 6 h. The DNA complexes were eluted using 0.2 M glycine. After crosslink reversal and purification, the samples were ready for PCR. Primers: L1: tcttcctcagacgtttccacgg, L2: tagaaaatgttagacacttgctggc, L3: actcccagaccacgagactt, L4: tcctgaggaggtaccaagaca.

### Western blot

Cells were harvested at the indicated times and rinsed twice with PBS. Cell extracts were prepared with lysis buffer and centrifuged at 12,000 rpm for 30 min at 4 °C. Protein samples were separated by sodium dodecyl sulfate-polyacrylamide gel electrophoresis (SDS-PAGE) in 7.5% (wt/vol) polyacrylamide gels and transferred to polyvinylidene fluoride membranes. After blocking with 5% BSA for 1 h at room temperature, the membrane was incubated with 2.5 μg/ml antibody in 5% BSA overnight at 4 °C. The membrane was then incubated with a secondary antibody conjugated to a fluorescent tag (Invitrogen). The band signals were visualized and quantified using the Odyssey Infrared Imaging System (LI-COR, USA). The following antibodies were used: anti-pRB 1:1000 (ab226979, Abcam, USA), anti-CTCF 1:500 (ab128873, Abcam, USA), anti-H3K27me3 1:500 (ab6002, Abcam, USA), and β-actin 1:5000 (A5316, Sigma-Aldrich).

### CRISPR/Cas9-mediated deletions

Four small gRNAs (sgRNAs) were cloned separately into lenti-Guide-Puro plasmids. To delete the 1.4 kb *RB1-S* region from the genome, RB44 cells were transfected with plasmids containing gRNAs (and Cas9) targeting the left and right side of the region to be deleted. Colonies were derived from single cells and tested for the loss of the targeted region. The control group was transfected with gRNA-empty vector using Lipofectamine 2000 (Invitrogen, Carlsbad, CA, USA) according to the manufacturer’s instructions.

### Immunofluorescence

The immunofluorescence assay was performed as previously described^39^. Human retinoblastoma tissues and normal eye tissues were incubated with anti-H3K27me3 (AB6002, Abcam, USA) antibodies at 4 °C overnight. Thereafter, the slides were incubated with the appropriate secondary antibodies for 30 min, and nuclei were counterstained with DAPI (Sigma-Aldrich) for 1 h. Digital images were obtained with a confocal microscope. Relative level of H3K27me3 was determined by comparing the fluorescence intensity of the target antibody with that of DAPI.

### CCK8 cell viability assay

For CCK8 assay, cells were seeded into a flat-bottomed 96-well culture plate at 2000 cells per well with 100 μl culture medium. In brief, 10 μl of CCK8 (Dojindo, Japan) solution was added to each well. The samples were incubated for 4 h; then the absorbance was measured at 450 nm in a microplate reader (Varioskan Flash; Thermo, USA) for 4 consecutive days.

### Plate colony formation assay

A plate colony formation assay was performed in six-well plates. A total of 1000 cells were suspended in 2.0 ml of complete medium and seeded into each well. The cells were cultured with complete medium for 10 days. For quantification, the colonies grown in plates were stained with 1% crystal violet and then photographed.

### Tumor xenograft model and in nude mice

Animal protocols were approved by the Animal Care and Use Committee at Shanghai Jiao Tong Medical College. Male 3-week-old nude mice were deeply anesthetized. To build orthotopic xenograft models, RB44 and RB44-*RB1-S*-deletion cells in a 4 μl sterile saline solution were injected into the sub retina and vitreous chamber of each eye through the sclera using a Hamilton syringe. After the injection, the eyes were treated with antibiotic eye drops. To build subcutaneous xenograft models, IM9 cells in a 0.2 ml volume of sterile saline solution were subcutaneously injected into the right flank. All mice were cervically dislocated 49 days after implantation for the tumor formation analysis.

### Statistics and reproducibility

All of the experiments were performed in triplicate, and the data were expressed as the mean ± standard deviation (SD, Error bars). The comparative threshold cycle method was applied in the quantitative real-time PCR assay according to the ΔΔ threshold cycle method. The differences between two groups were analyzed with the unpaired two-sided Student’s *t*-test. *p* value of less than 0.01 or 0.001 was considered statistically significant and is indicated with double or triplicate asterisks, as described in the figure legends.

### Reporting summary

Further information on research design is available in the Nature Research Reporting Summary linked to this article.

## Supplementary information

Supplementary Information

Description of Additional Supplementary Files

Supplementary Data 1

Supplementary Data 2

Reporting Summary

## Data Availability

All uncropped and unedited blot/gel images are available in Supplementary Fig. 5. All source data of all *RB1* exons in RB44 cell is available in Supplementary Data 1, these data show all original sequences of 27 exons of *RB1* gene in RB44 cells for aligning with existed *RB1* NCBI Reference Sequence: NM_000321.3. All source data underlying the graphs and charts presented in the main figures are available in Supplementary Data 2.
