# Supplementary material for: Interruption of aberrant chromatin looping is required for regenerating RB1 function and suppressing tumorigenesis
Source: Commun Biol. 2022 Sep 29;5:1036. doi: 10.1038/s42003-022-04007-2 (PMC9522773; doi:10.1038/s42003-022-04007-2)
Supplement: Supplementary file 1 — Supplementary Information [file 42003_2022_4007_MOESM1_ESM.pdf]

Supplementary Figure Legends

Figure S1 The peaks of protein binding across the entire RB1 locus in UCSC genome browser.

a: The binding peaks of CTCF, H3K27me3 and H3K4me3 were illustrated at the entire *RB1* locus. Arrow show the direction of primer, E1-14 represent the primer name.

Figure S2 The chromatin looping in HDF and RB44 cells.

a: The intrachromosomal interactions was detected between E5 and Ec in HDF cells. HDF: human fibroblasts. Ec site: intron 20 adjacent to exon 21 of the RB1 gene.

b: The 3C products were confirmed by DNA sequencing. The 3C products derived from the RB1 promoter E5-Ec interaction were cloned and sequenced. The 3C products contained the EcoRI site that was flanked on intron 20 adjacent to exon 21.

Figure S3 Pairs of forward-reverse CTCF located in E5 and E7 regions.

a: The 19 bp motif of CBS and frequency matrix from JASPAR (https://jaspar.genereg.net/matrix/MA0139.1/). The conserved C5, G10 and G13 (shown in yellow) are necessary reference for judging the existence of CBS. And the A9 (shown in blue) represents the forward orientation of CBS ("+", forward; "-", reverse).

b: The forward CBSs predicted in the E5 promoter region. Left: E5 was shown in purple, EcoRI cutting site was shown in red, and CBSs was shown in grey. Right: the sequence of six forward CBSs; yellow, the conserved nucleobase in CBS; blue, the orientation reference of CBS.

c: The reverse CBSs predicted in the E5 suppressor region. Left: E7 was shown in green,

EcoRI cutting site was shown in red, and CBSs was shown in grey. Right: the sequence of five reverse CBSs; yellow, the conserved nucleobase in CBS; blue, the orientation reference of CBS.

Figure S4 SMC1 is not involved in the formation of RB1-P-S loop

a: qRT-PCR showed that SMC1 was silenced by shRNAs transfection in RNA level.

b: Western blot was performed to detect SMC1 protein expression after shRNA transfection. Normal cells: RPE cell.

c-d: A 3C assay was performed to detect the existence of chromosomal looping after SMC1 knockdown. Result showed that SMC1-knockdown did not abolish the intrachromosomal loop between RB1 promoter and suppressor.

Figure S5 All uncropped and unedited blot/gel images.

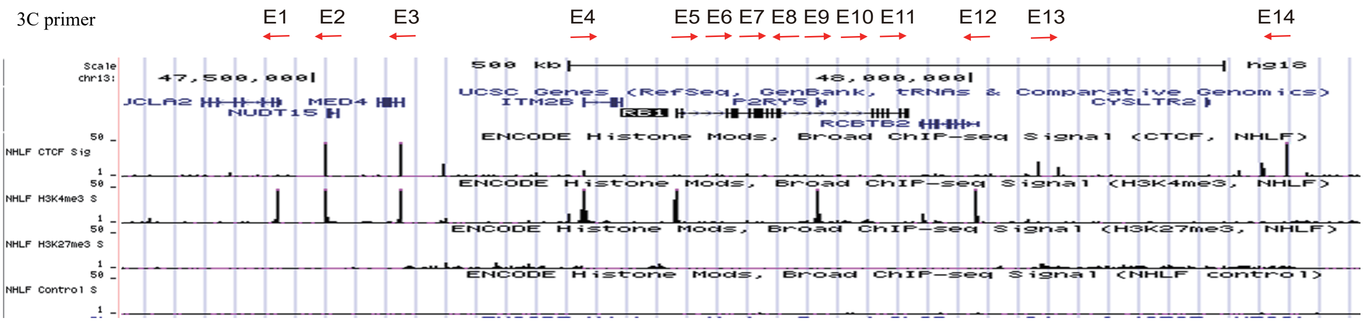

Supplementary Figure 1 The peaks of protein binding across the entire RB1 locus in UCSC genome browser

a

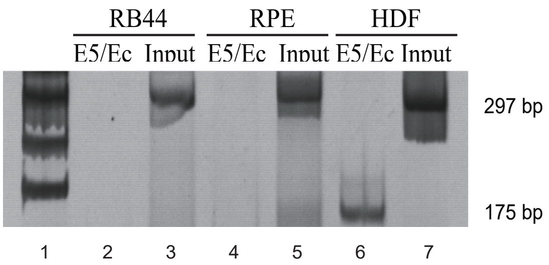

b

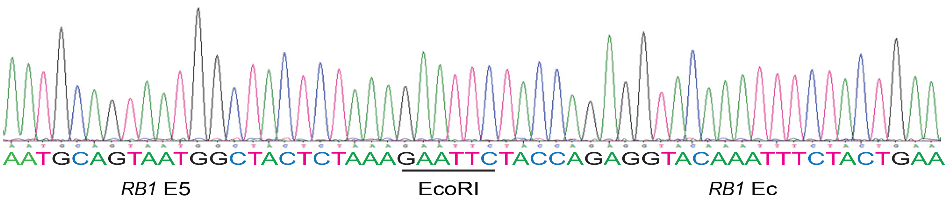

Supplementary Figure 2 The chromatin looping in HDF cells.

a

CBS motif

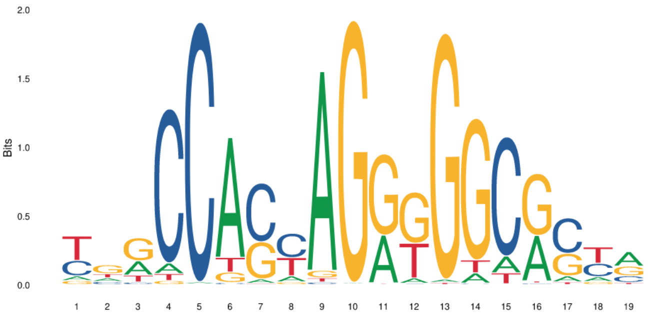

Frequency matrix

	1	2	3	4	5	6	7	8	9	10	11	12	13	14	15	16	17	18	19	
A [	87	167	281	56	8	744	40	107	851	5	333	54	12	56	104	372	82	117	402	]
C [	291	145	49	800	903	13	528	433	11	0	3	12	0	8	733	13	482	322	181	]
G [	76	414	449	21	0	65	334	48	32	903	566	504	890	775	5	507	307	73	266	]
T [	459	187	134	36	2	91	11	324	18	3	9	341	8	71	67	17	37	396	59	]

b

E5 region

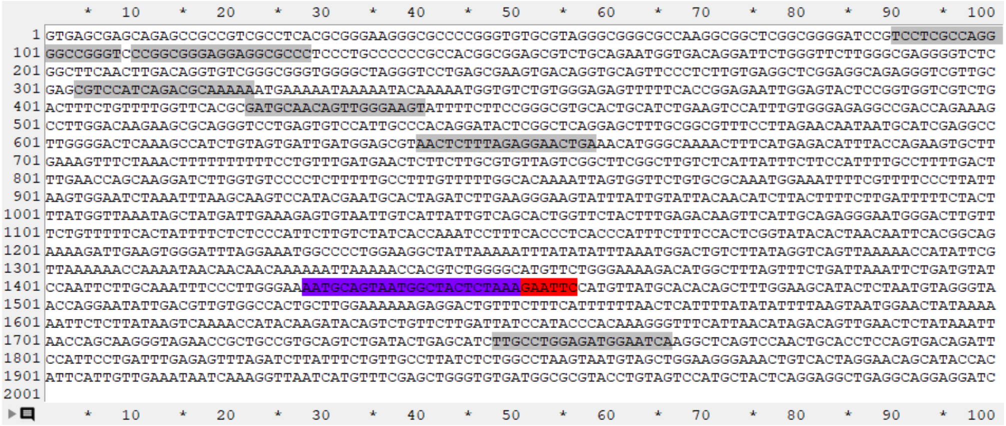

CBS (5'→3')	orientation
TCCTCGCCAGGGGCCGGGT	+
CCGGCGGGAGGAGGCGCCC	+
CGTCCATCAGACGCAAAAA	+
GATGCAACAGTTGGGAAGT	+
AACTCTTTAGAGGAACTGA	+
TTGCCTGGAGATGGAATCA	+

c

E7 region

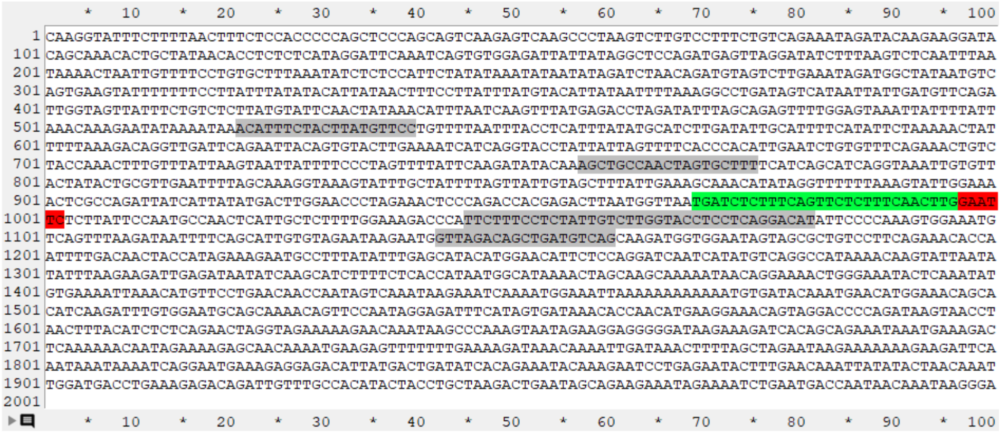

CBS (5'→3')	orientation
ACATTTCTACTTATGTTCC	−
AGCTGCCAACTAGTGCTTT	−
TTCTTTCCTCTATTGTCTT	−
TGGTACCTCCTCAGGACAT	−
GTTAGACAGCTGATGTCAG	−

Supplementary Figure 3 Pairs of forward-reverse CTCF located in E5 and E7 regions.

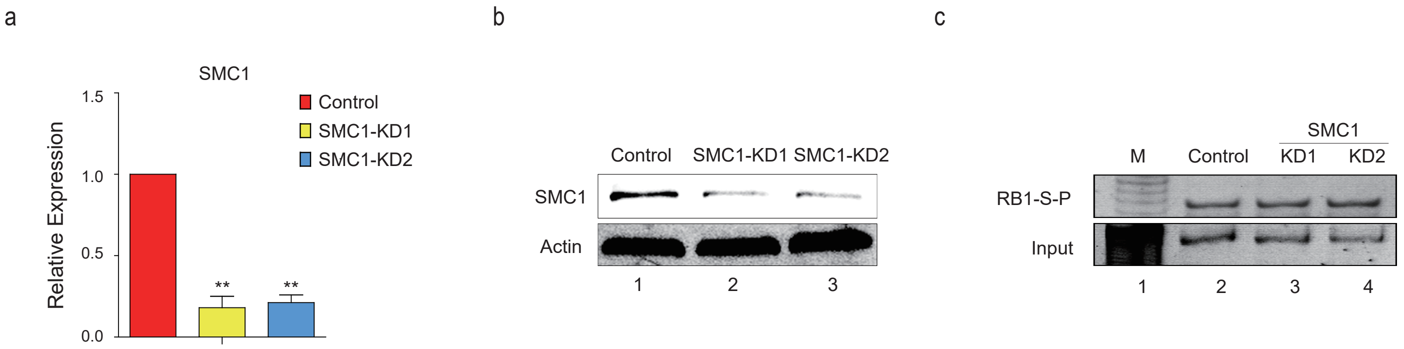

Supplementary Figure 4 SMC1 is not involved in the formation of RB1-P-S loop

Supplementary Figure 5 All uncropped and unedited blot/gel images.

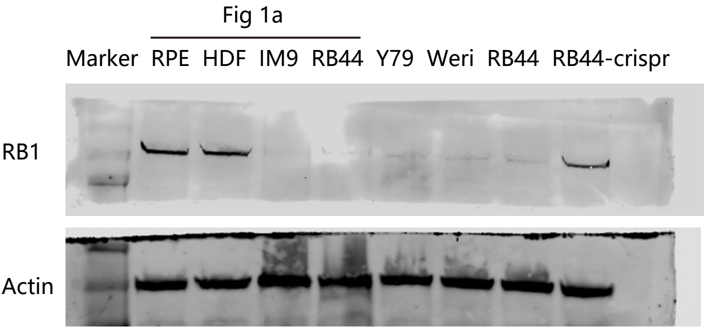

Fig 1c

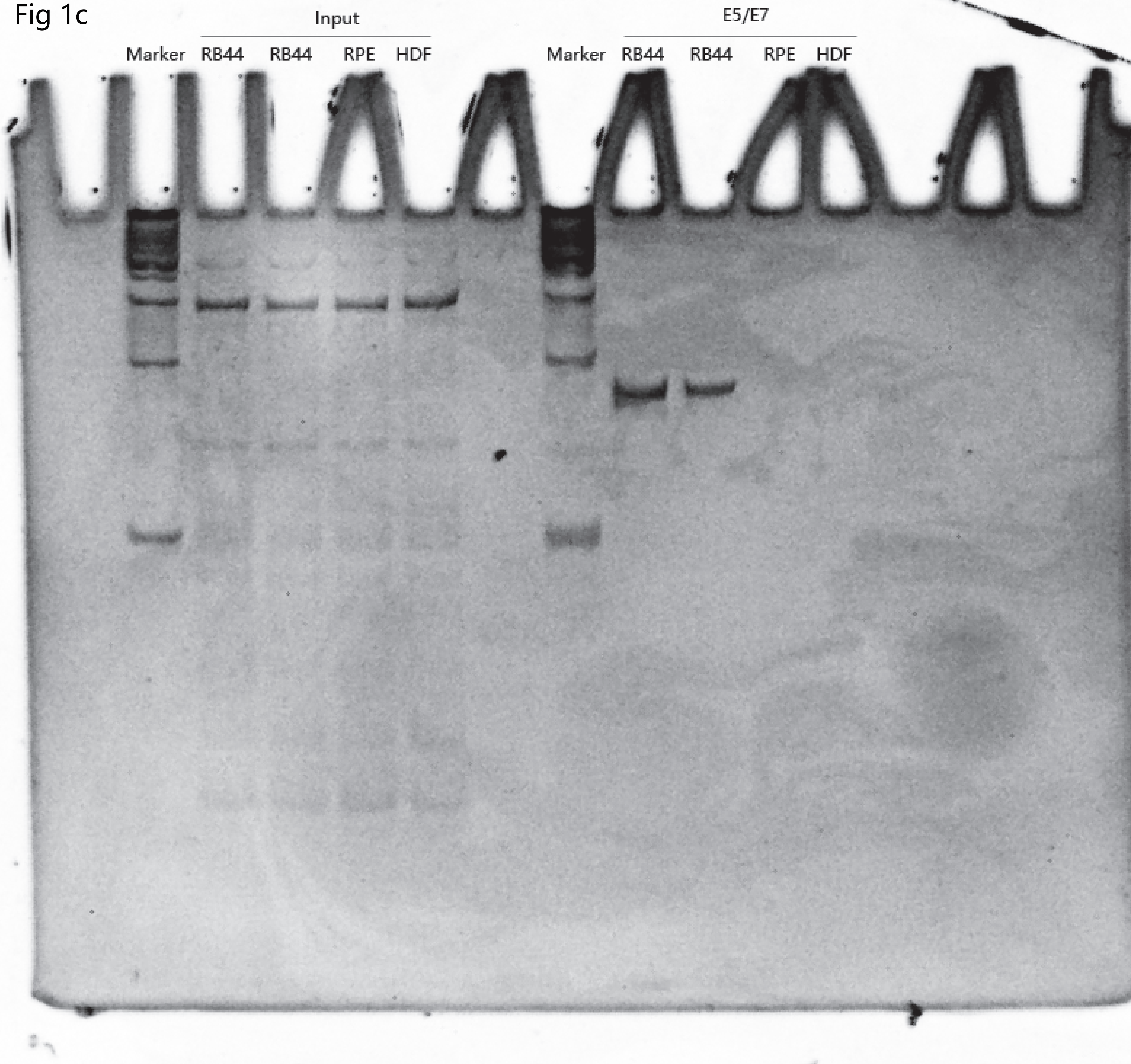

Fig 1e

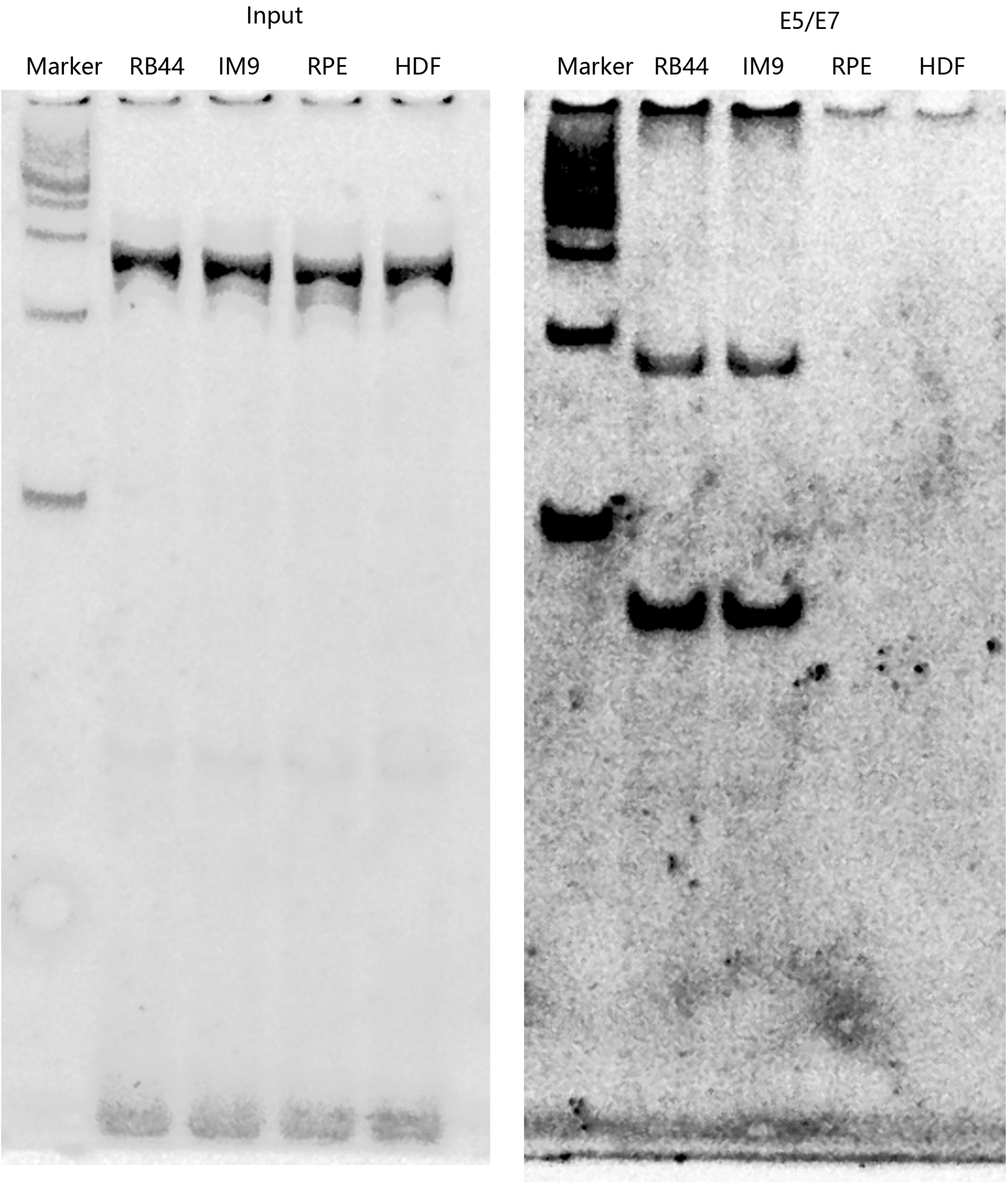

Fig 2b

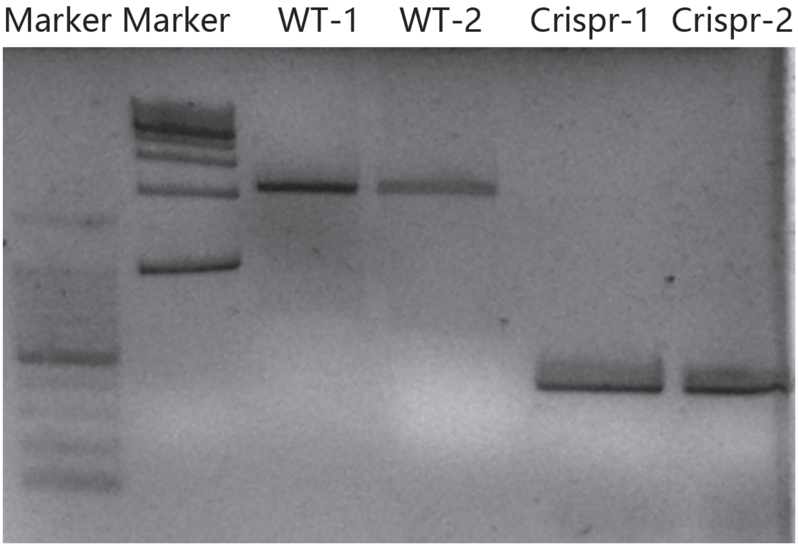

Fig 2d

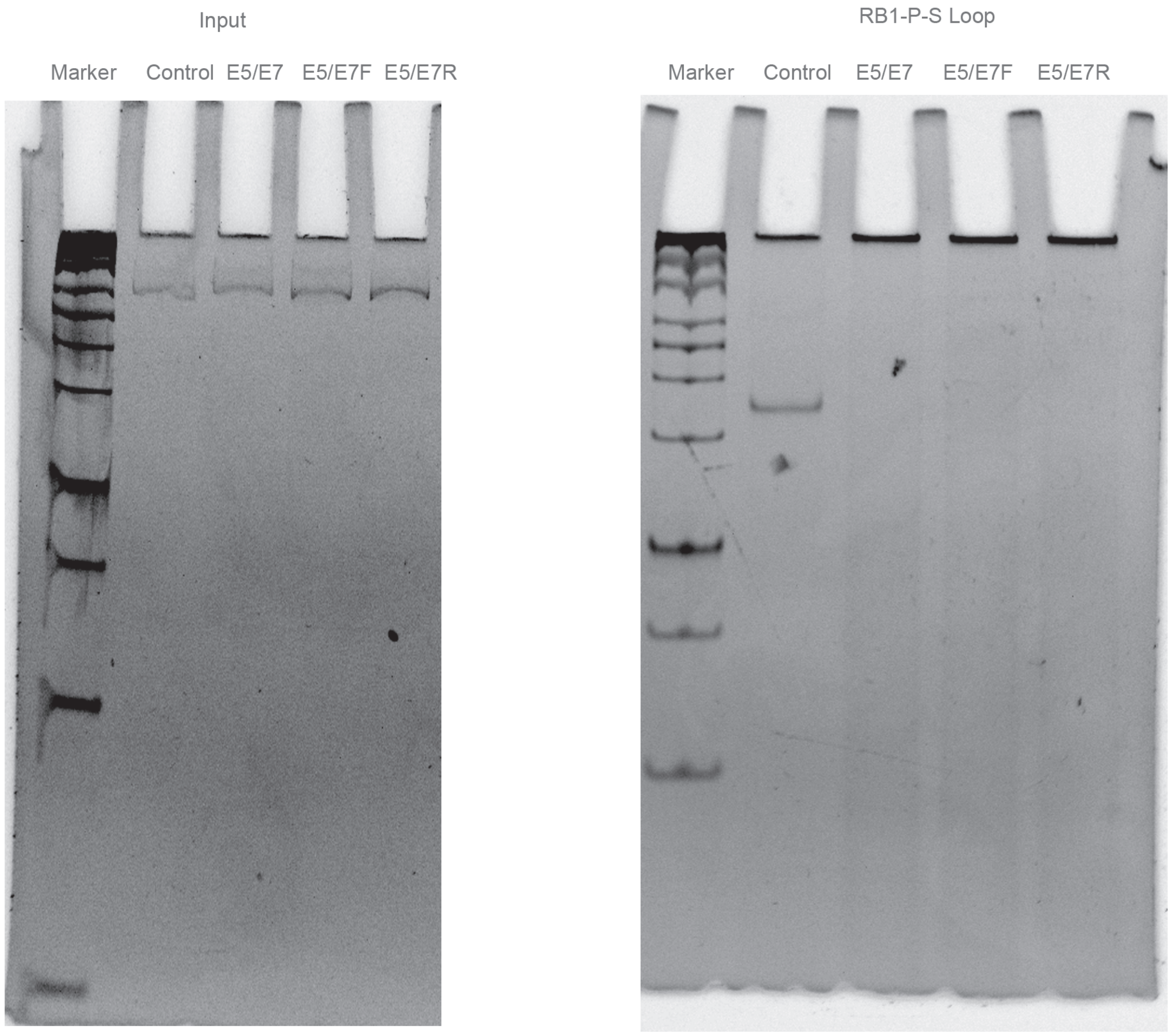
Input
Marker
Control
E5/E7
E5/E7F
E5/E7R
RB1-P-S Loop
Marker
Control
E5/E7
E5/E7F
E5/E7R

Fig 2e

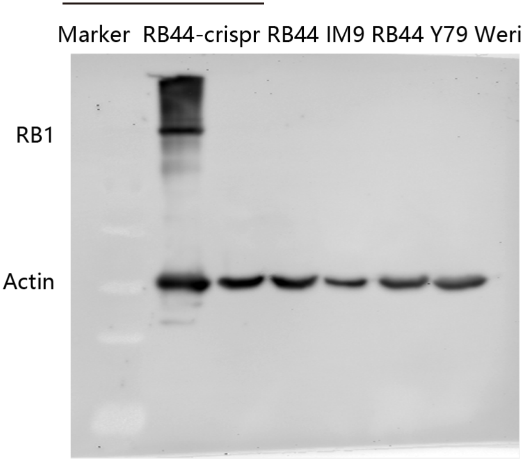

Fig 3a normal panel

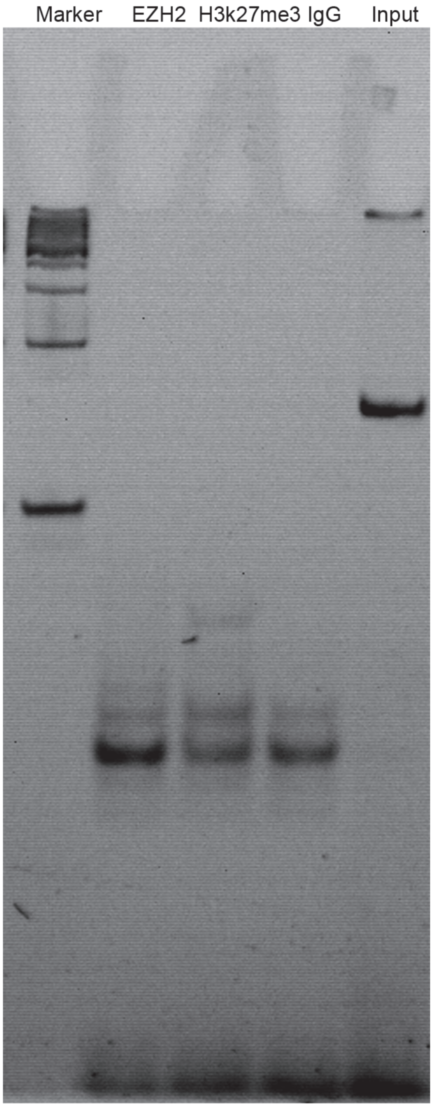

RB1-P

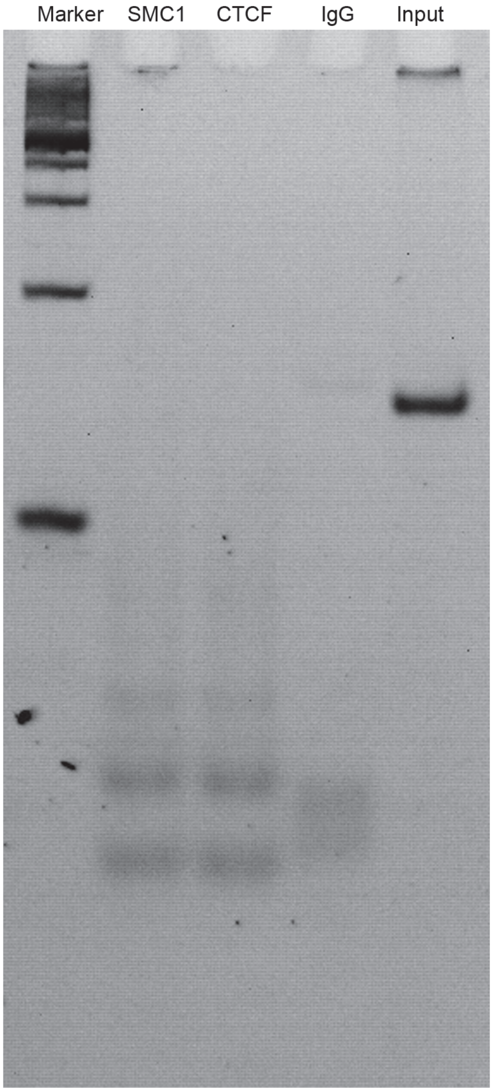

RB1-P

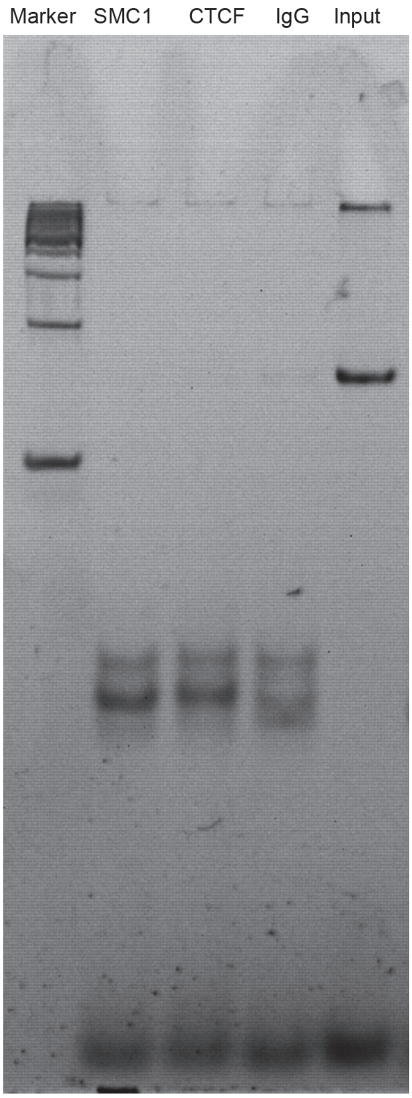

RB1-S

Fig 3a RB44 panel

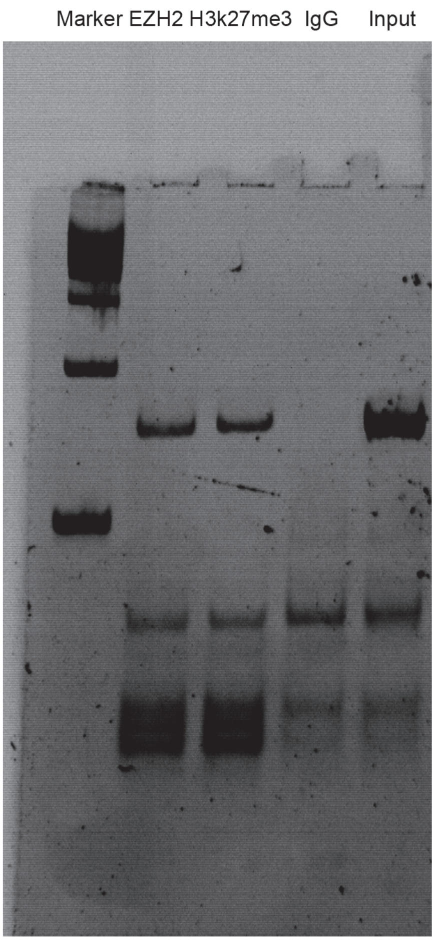

RB1-P

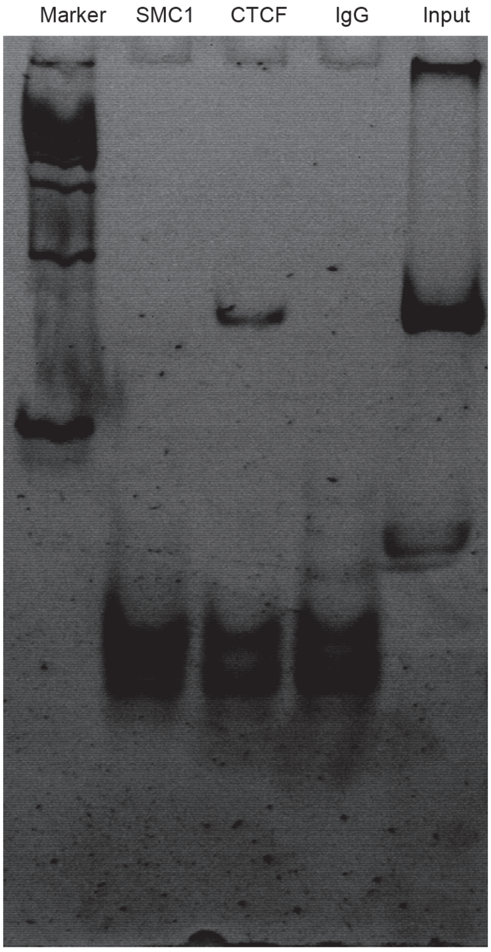

RB1-P

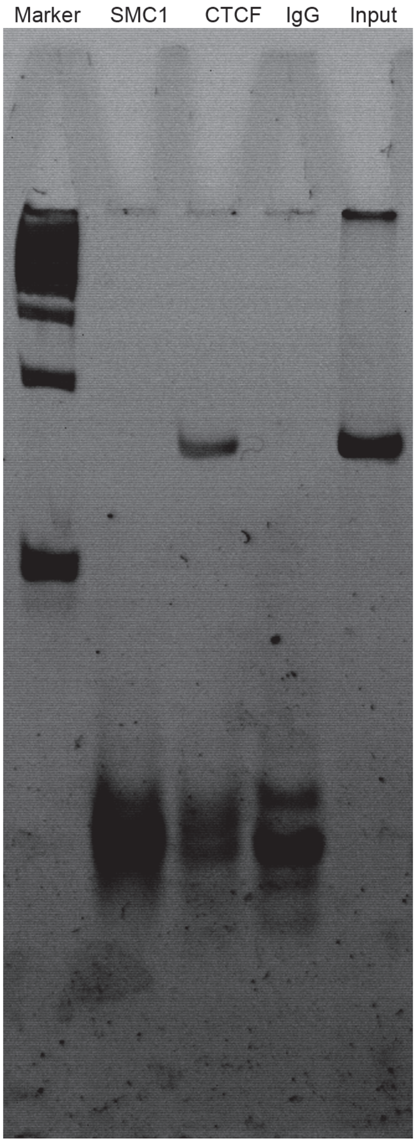

RB1-S

Fig 3a RB44-crispr Panel

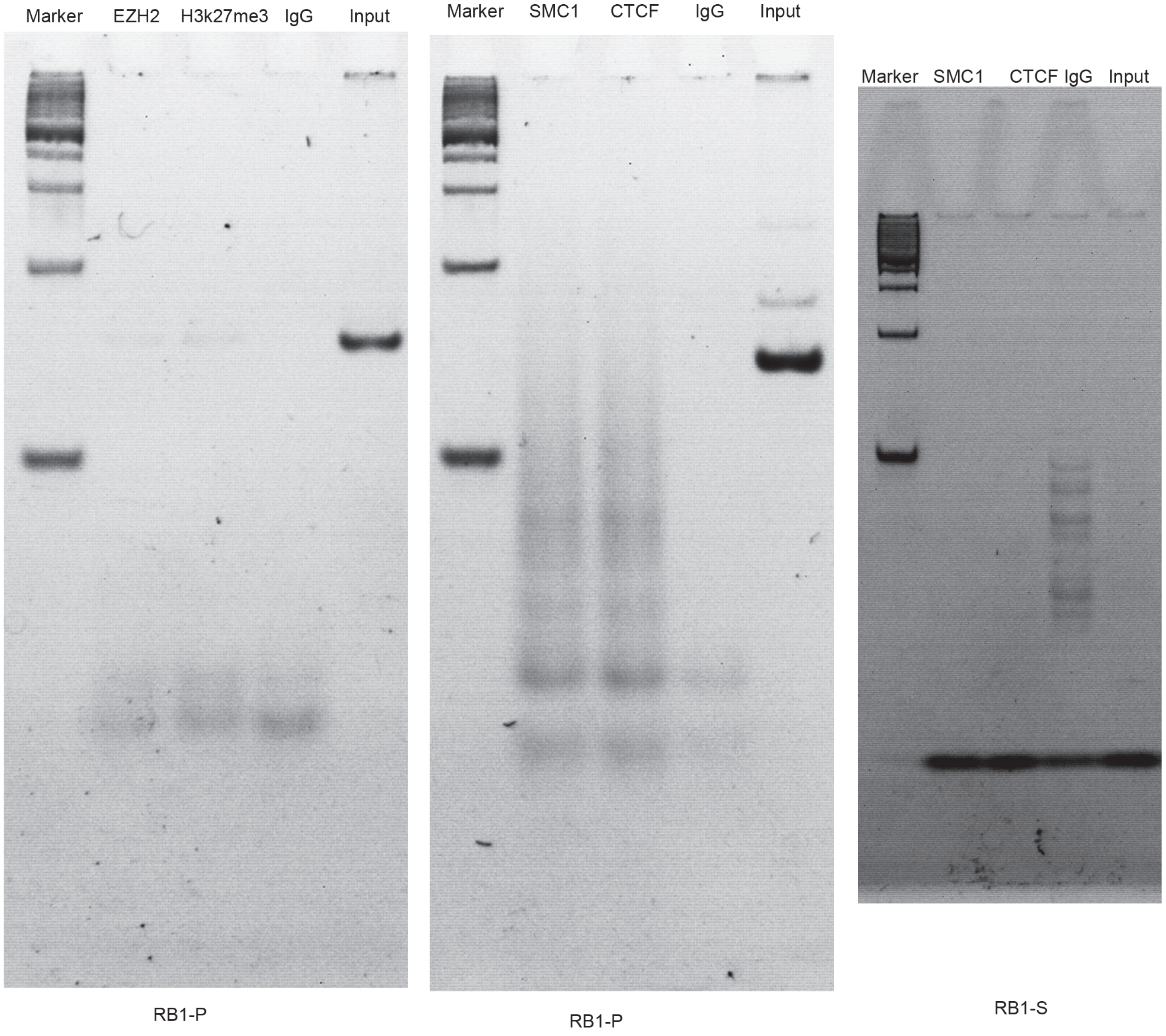

Fig 4b

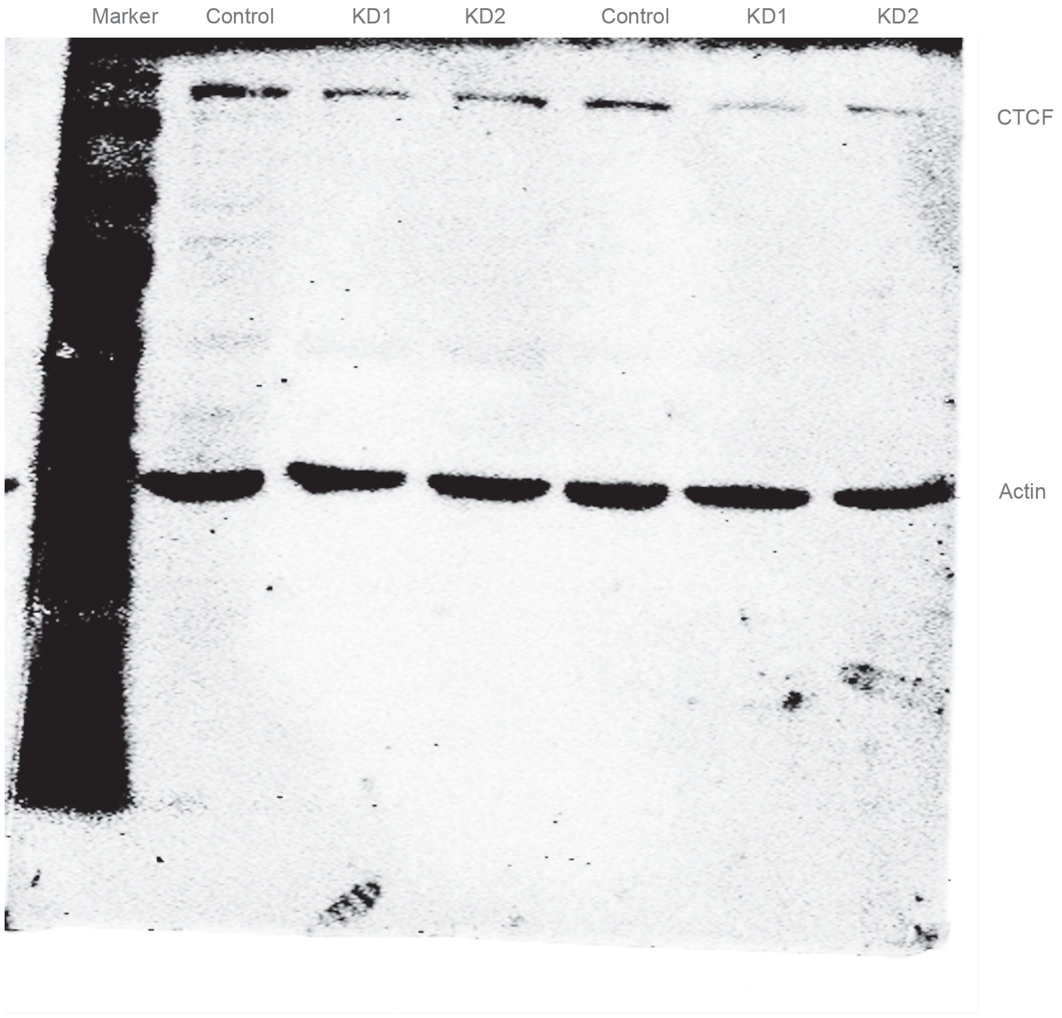

Fig 4c

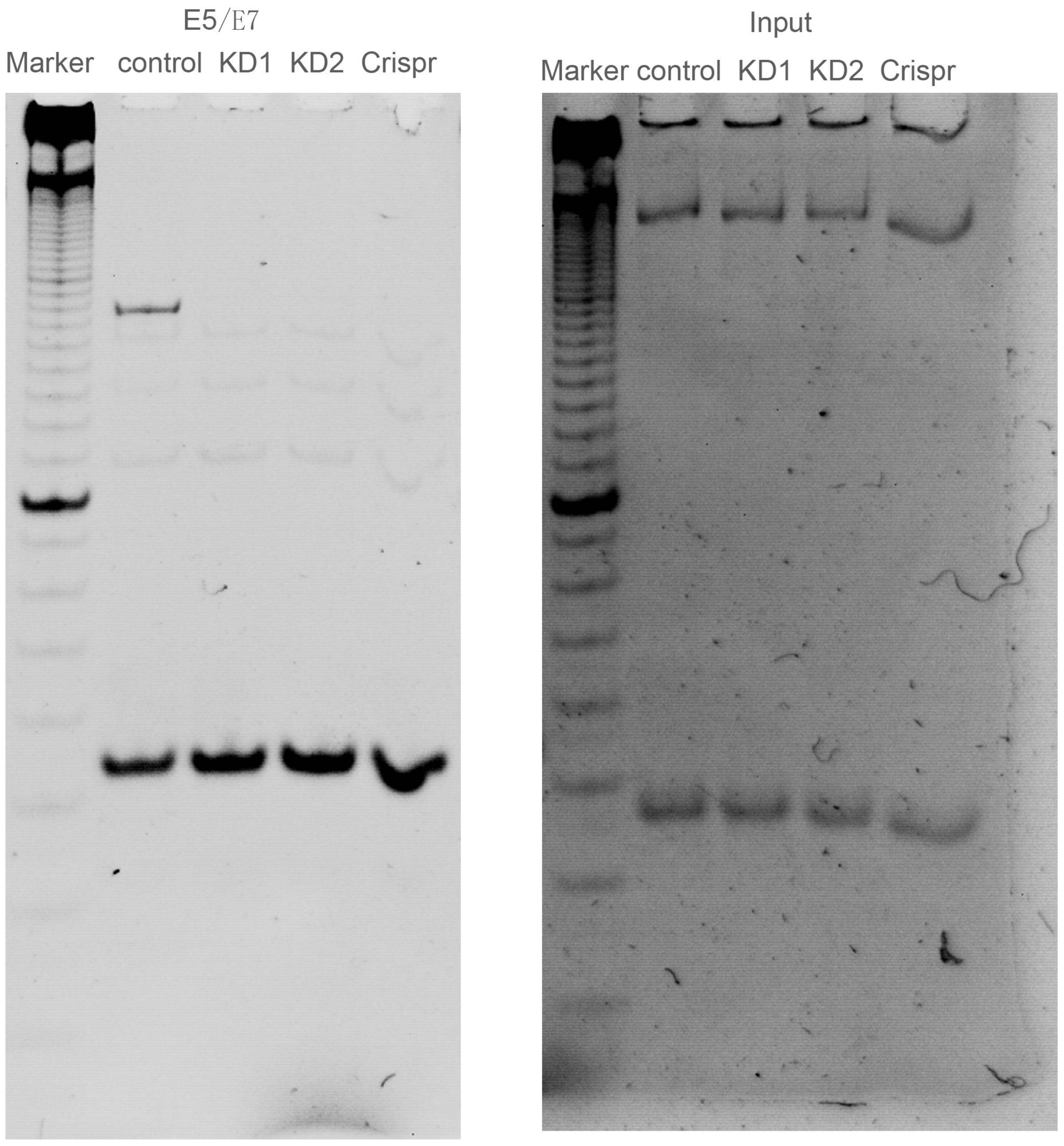
E5/E7
Marker control KD1 KD2 Crispr
Input
Marker control KD1 KD2 Crispr

Fig 4e 1st and 2nd panel

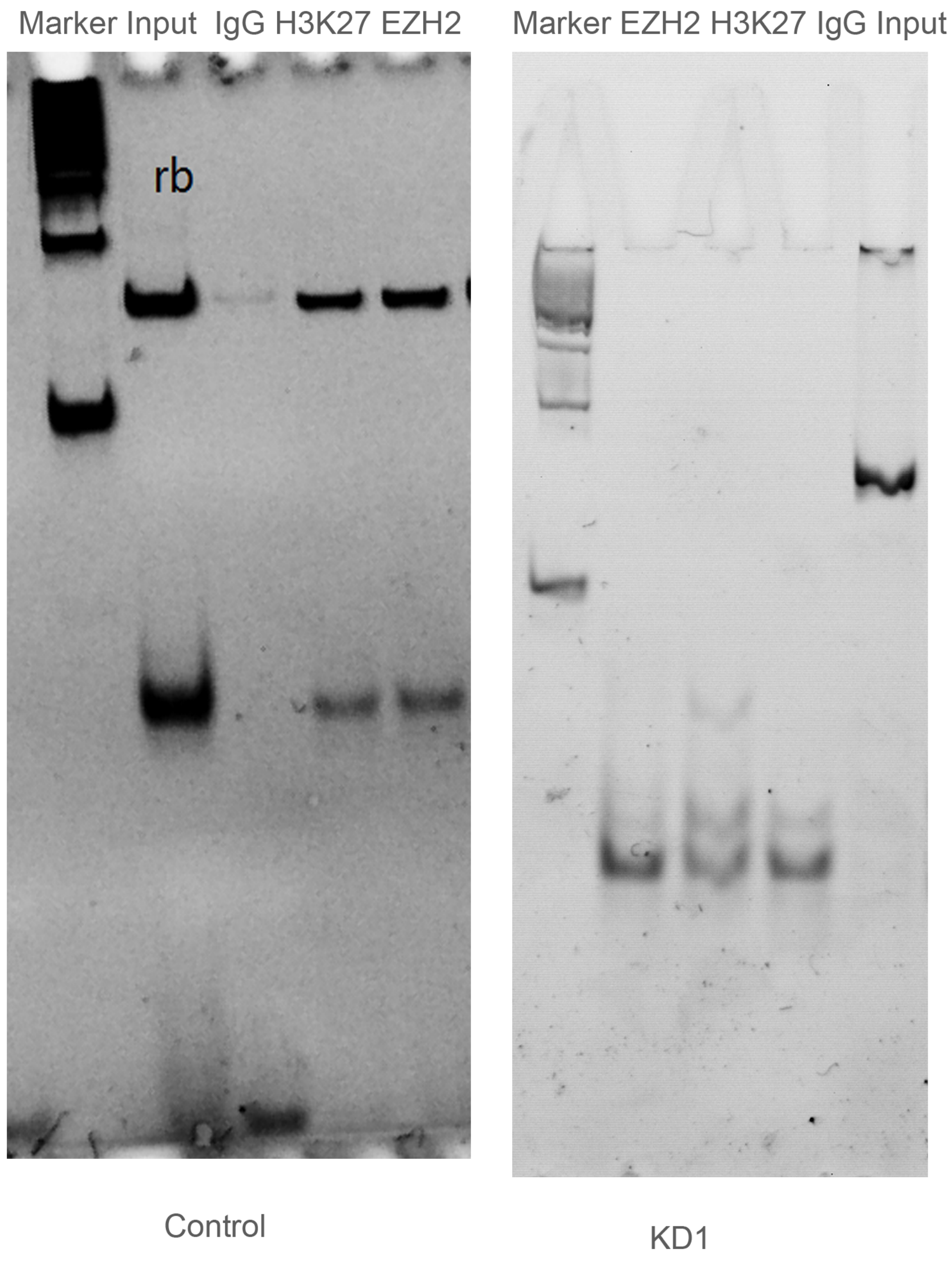

Fig 4e 3rd and 4th panel

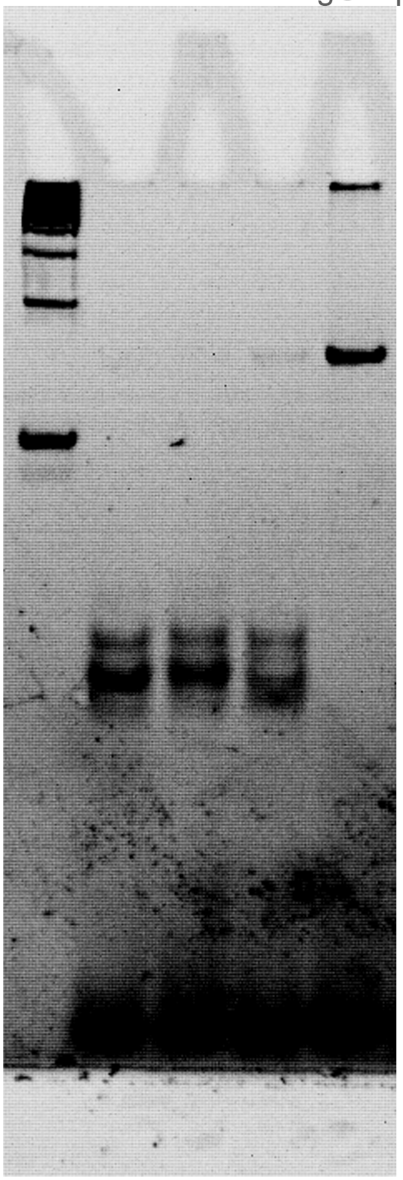

KD2

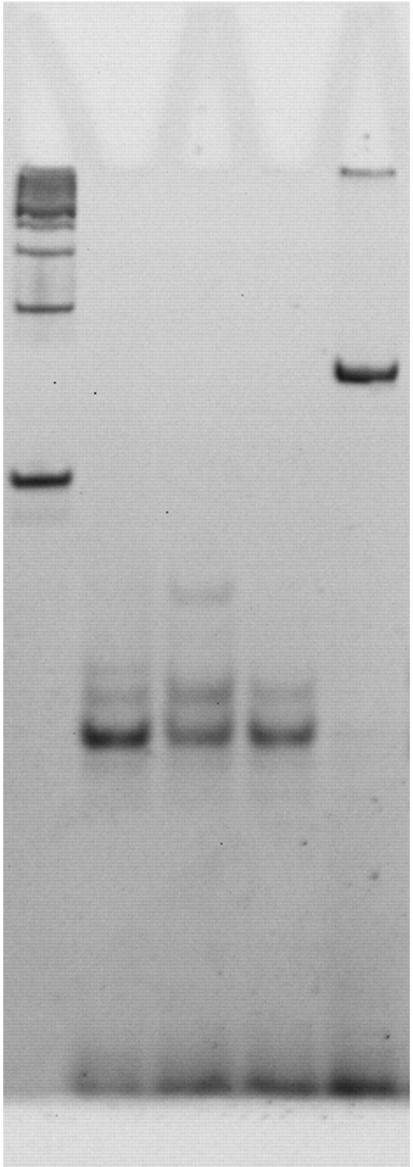

Normal

Fig 4g

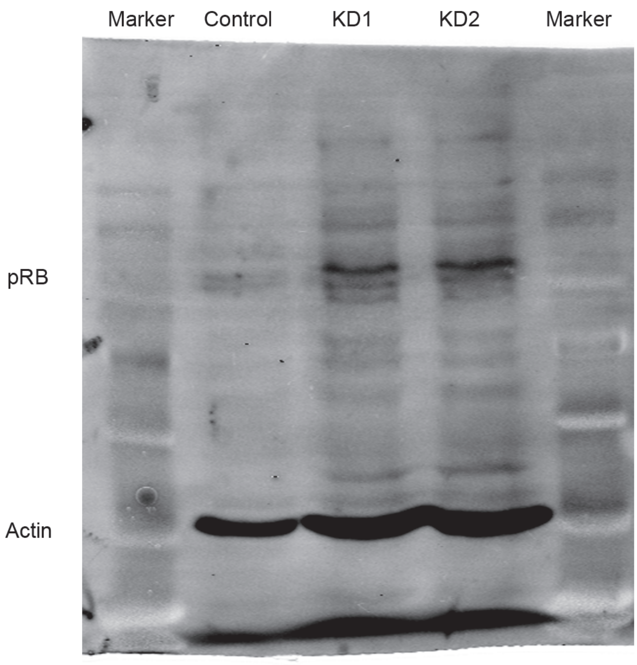

Fig 5a

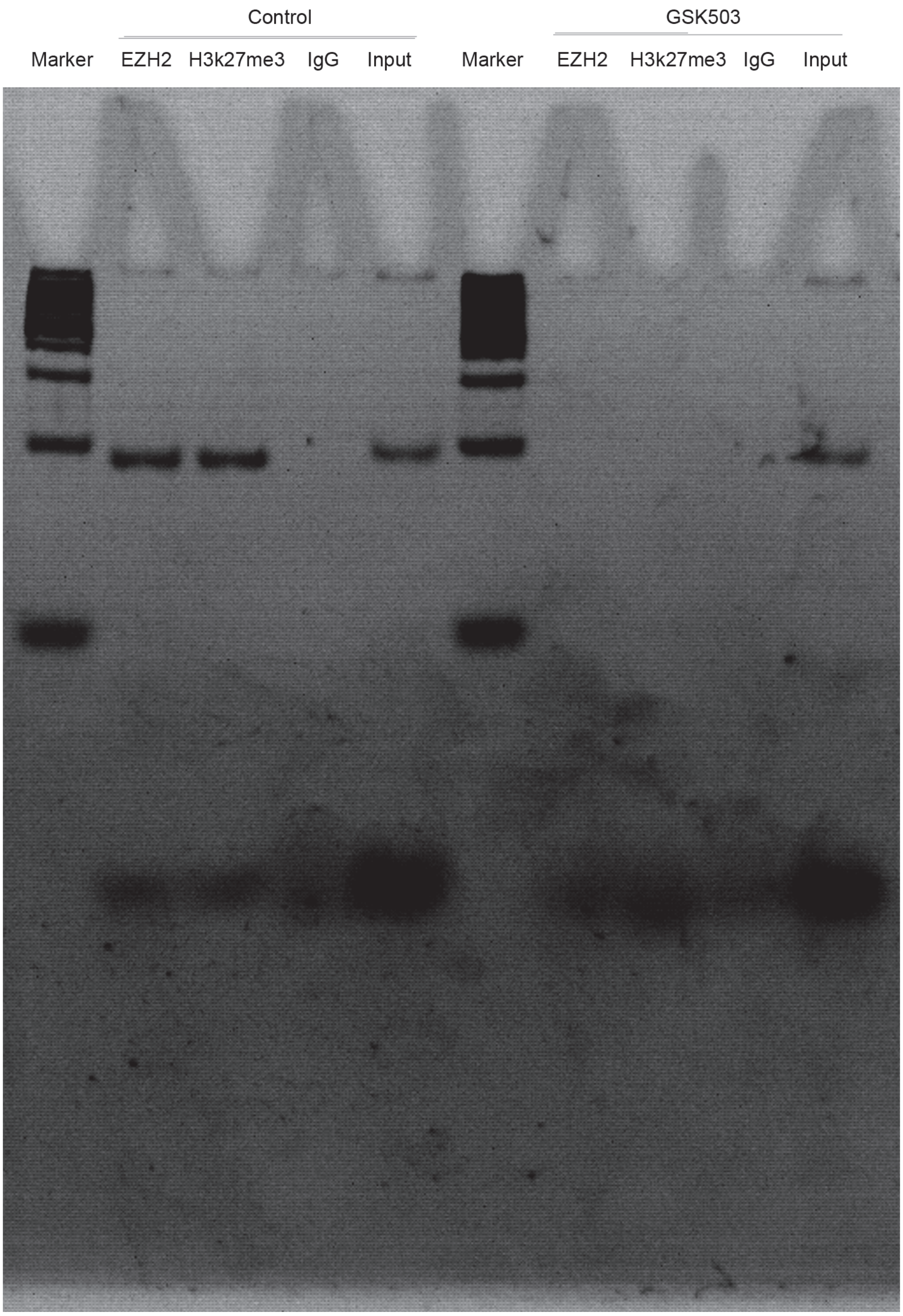

Control
GSK503
Marker
EZH2
H3k27me3
IgG
Input
Marker
EZH2
H3k27me3
IgG
Input

Fig 5c

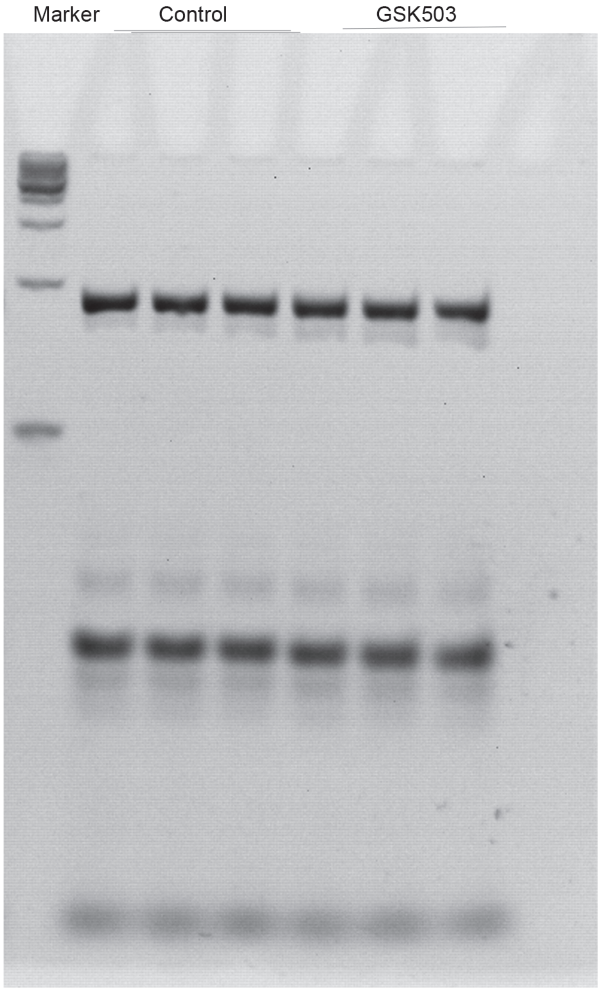

Input

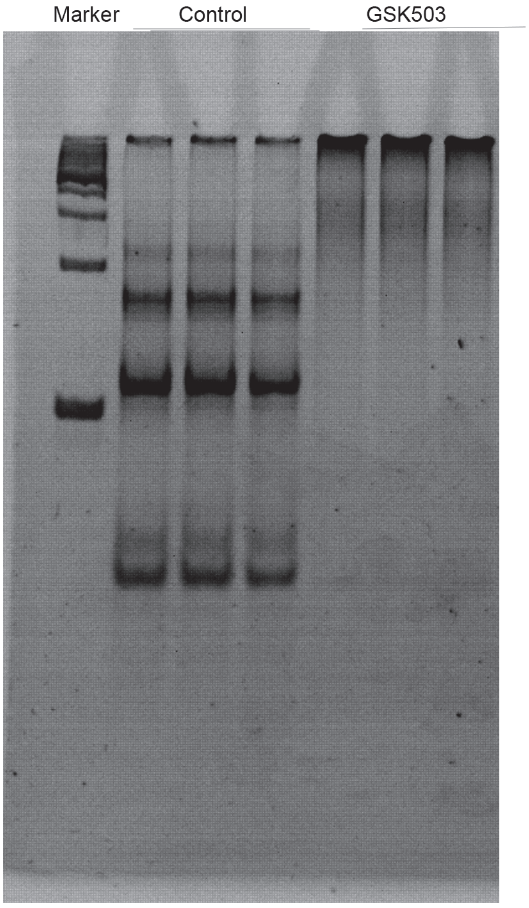

Fig 5e

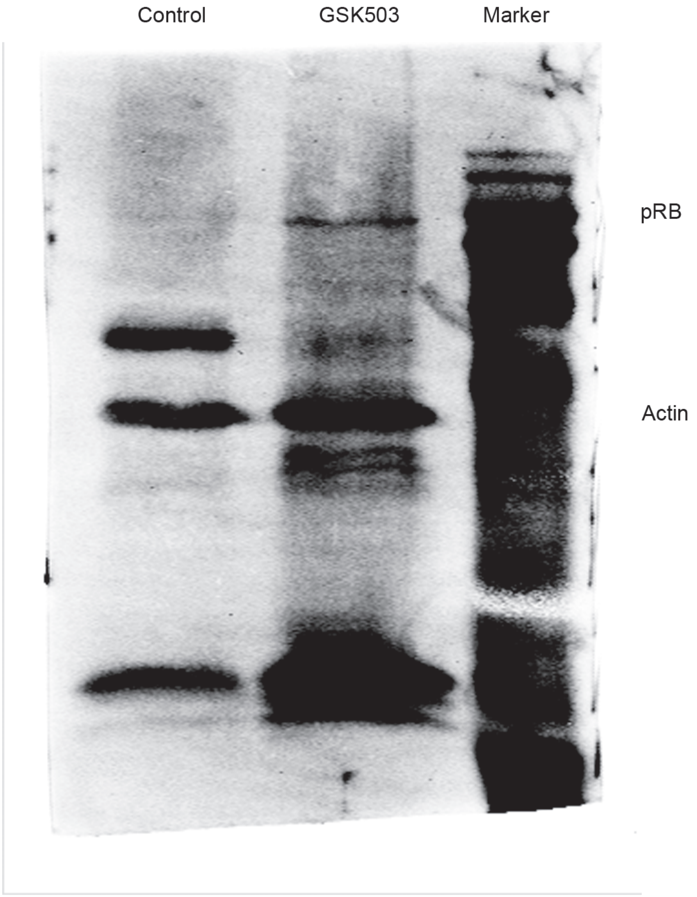

Supplementary table 1. Exon sequencing

RB1	Forward (5′-3′)	Reverse (5′-3′)	Length (bp)	Exon Mutation
exon 1	CTCAGGGGACGTTGAAAT	GCCCAAGAACCCAGAATC	651	None
exon 2	AACAAGTATGTACTGAATCAATTTG	CTATCTTTCAATTTTTGTATAGTGA	731	None
exon 3	CAGTTTTAACATAGTATCCAGTGTG	GGAAAATCCAGAATTCGTTTCC	560	None
exon 4	TGATGTAGAGCTGATAATCTTTTG	AATTCCCAGAATCTAATTGTGAAC	641	None
exon 5	AACTACTATGACTTCTAAATTACG	CTTAATTTATGAAGTAGCCTGCTA	582	None
exon 6	CTGGAAAACTTTCTTTCAGTGATA	GGAATTTAGTCCAAAGGAATGCC	688	None
exon 7	CTCTACCCTGCGATTTTC	GTCTCCCAAACCTCCATT	423	None
exon 8	TTGGCTATTTCCATGCCTTC	TTCCAGAGTGAGGGAGCTACT	621	None
exon 9	GGTGGAAGGATGGCTTGA	CCTCCCTCCACAGTCTCAA	391	None
exon 10	GCCTCTGTGTGCTGAGAGATGTA	AATGATATCTAAAGGTCACTAAGC	453	None
exon 11	GATTTTATGAGACAACAGAAGCA	ATCTGAAACACTATAAAGCCATG	244	None
exon 12	CAATACCATTTTGTTGCCAGTTT	TGCAAGGAAGAATGGTGAGC	647	None
exon 13	TGAGGCTCTTTCTGATGGATGG	TGGGGTGGGAGGTAGTTTTCC	595	None
exon 14	TCTTGAGCCCAGGAGTGT	CAGGATGATCTTGATGCC	305	None
exon 15/16	CAATGCTGACACAAATAAGGTT	AAGAAACACACCACATTTTAACT	321	None
exon 17	AGCTCAAGGGTTAATATTTCATAA	AATTTGTTAGCCATATGCACATG	302	None
exon 18	ATGTACCTGGGAAAATTATGCTT	CTTTATAGAATGTTACATTGCAC	258	None
exon 19	GGCAGTAATCCCCAGGAA	ACCCAGTCAGCCTAGTTTC	422	None
exon 20	CTGGGGGAAAGAAAAGAGTGG	GAGGAGAGAAGGTGAAGTGCT	328	None
exon 21	TGAGCCTTGGTGATTTGC	TTGATTTGCAAGGCTGCAT	677	None
exon 22	CTGCTGCTGCCTGGCTAT	AGGGCTTCGAGGAATGTG	498	None
exon 23	CAGTATGCTTCCACCAGG	TCGGCCATCTTGCGTTGC	473	None
exon 24	CTTGCCTTTGCCCTCCCT	TGCCTGGATGAGGTGTTTG	418	None
exon 25	TGGGTGTTTAATTGGGGATG	TGAGCCATTCTCACAACTTCC	479	None
exon 26	GCATACCAACATTCAGTGAAG	TGTTGAATGTGGTCAAGC	643	None
exon 27	TGCAAGGTCCTGAGCGCCAT	GAGAGACAATGAATCCAGAGGTG	458	None

Supplementary table 2. PCR primers and amplification details for 3C products

Region	Primer(5′-3′)
E1	tgcttagaacactgcccaccatgtc
E1-2	tgcttagaacactgcccaccatgtcaat
E2	agatgtggtggtatctgcctatggt
E2-2	agatgtggtggtatctgcctatggttcc
E3	actcgtgaggctgaggcaggagaat
E3-2	actcgtgaggctgaggcaggagaatggc
E4	atgtggtaggactgcaggacagaac
E4-2	atgtggtaggactgcaggacagaacaga
E5	gcatgttctgggaaaagacatggct
E5-2	gcatgttctgggaaaagacatggcttta
E6	ccacttctctgttgacagacacata
E6-2	ccacttctctgttgacagacacataggt
E7	cctagaaactcccagaccacgagac
E7-2	cctagaaactcccagaccacgagactta
E8	cccatgctgattgctctttaacaga
E8-2	cccatgctgattgctctttaacagagca
E9	gggctgttgttgtcatacattttgc
E9-2	gggctgttgttgtcatacattttgcttc
E10	caccctcccaagagtaaaccaggaa
E10-2	caccctcccaagagtaaaccaggaagaa
E11	ccatgttaaggtccagaatgactgg
E11-2	ccatgttaaggtccagaatgactggcaa
E12	tgtctatggctaggaccagtcacat
E12-2	tgtctatggctaggaccagtcacatggc
E13	ctgggtatgaggaatgtggcctgga
E13-2	ctgggtatgaggaatgtggcctggagat
E14	cacttcgggaggccaagatgagagg
E14-2	cacttcgggaggccaagatgagaggata
Ec	agttagggaggagtcccttcctttc
Ec-2	agttagggaggagtcccttcctttcaat
Input F	ttctctgggctgatgctcctttctt
Input F-2	ttctctgggctgatgctcctttcttgtc
Input R	tcccaggcattcctgttgaggt
Input R-2	tcccaggcattcctgttgaggtgag

Supplementary table 3. Specific 3C primers of RB1-S region

Region	Primer(5′-3′)
E7	cctagaaactcccagaccacgagac
E7-2	cctagaaactcccagaccacgagactta
E7F	gatacagcaaacactgctataacacc
E7F-2	gatacagcaaacactgctataacacctct
E7R	tctgagagatgtaaagttaggttact
E7R-2	tctgagagatgtaaagttaggttacttat

Supplementary table 4. Primer and shRNA sequences

	Sequence/ shRNA catalog number from Open Biosystems
Del-F:	CCTCTCCATTACTCCAGCAGC
Del-R:	GGCAAACAATCTGTCTCTTTCAGG
shCTCF	ATTACCAACTACTTTCTCTGC
shCTCF	ATGTAGATGTGTCTGTCTAC
shSMC1	H4 (V3LHS_637850)
shSMC1	H10 (V3LHS_637855)
