# Supplementary material for: Interruption of aberrant chromatin looping is required for regenerating RB1 function and suppressing tumorigenesis
Source: Commun Biol. 2022 Sep 29;5:1036. doi: 10.1038/s42003-022-04007-2 (PMC9522773; doi:10.1038/s42003-022-04007-2)
Supplement: Supplementary file 2 — Description of Additional Supplementary Files [file 42003_2022_4007_MOESM2_ESM.pdf]

Description of Additional Supplementary Files

File name: Supplementary Data 1

Description: The sequences of all RB1 exons in RB44 cells. These files (with .ab1 and .seq) show all original sequences of 27 exons of RB1 gene in RB44 cells for aligning with existed NCBI RB1 Reference Sequence: NM_000321.3. These files show exon sequences of single RB1 gene and is not High-throughput sequencing data. These files can be read in most biological softwares and is available in Supplementary Data 1.

File name: Supplementary Data 2.

Description: All source data underlying the graphs and charts presented in the main figures is available in Supplementary Data 2.
